# NMR-based metabonomics reveals the dynamic effect of electro-acupuncture on central nervous system in gastric mucosal lesions (GML) rats

**DOI:** 10.1186/s13020-022-00593-9

**Published:** 2022-03-21

**Authors:** Miaosen Huang, Yiwei Peng, Qida He, Linyu Lian, Yichen Wang, Longbin Zhang, Yuan Zhang, Jiacheng Shen, Zongbao Yang

**Affiliations:** 1 School of Medicine, Xiamen University, Xiamen, 361102 Fujian China; 2 College of Acupuncture and Moxibustion, Fujian University of Traditional Chinese Medicine, Fuzhou, 350122 China; 3 Faculty of Chinese Medicine and State Key Laboratory of Quality Research in Chinese Medicines, Macau University of Science and Technology, Taipa, 999078 Macau China; 4 Department of Acupuncture and Moxibustion, the Second Affiliated Hospital of Guangzhou University of Chinese Medicine, Guangzhou, 510405 Guangdong China; 5 Shanghai University of Traditional Chinese Medicine, Shanghai, 201203 Shanghai China

**Keywords:** Gastric mucosal lesions, Electro-acupuncture, NMR metabonomics, Dynamic expression

## Abstract

**Background:**

Gastric mucosal lesions (GML) are common in gastric diseases and seriously affect the quality of life. There are inevitable side effects in drug therapy. Acupuncture is an important part of traditional Chinese medicine. Electro-acupuncture (EA) has unique curative effect in treatment of GML. However, there are still few reports on the central mechanism of electro-acupuncture in treatment of GML. In this study, NMR metabonomics was used to explore the central metabolic change mechanism of electro-acupuncture in treatment of GML.

**Methods:**

SD rats were randomly divided into Control, GML and EA groups. According to different intervention time, each group was further divided into 3 subgroups. This study mainly established GML model rats by 75% ethanol. Dynamic expressions of metabolites in cerebral cortex and medulla were observed by 1D ^1^H Nuclear Magnetic Resonance (NMR) metabolomics, combined with gastric mucosal histopathological examination to evaluate the time-effect relationship of electro-acupuncture at Zusanli (ST36) and Liangmen (ST21) points for 1 day, 4 days and 7 days treatment of GML.

**Results:**

The results showed that the repair effect of electro-acupuncture on gastric mucosal injury was the most obvious in 4 days and stable in 7 days, and 4 days electro-acupuncture can effectively inhibit GML gastric mucosal inflammation and the expression of inflammatory cells. Meanwhile, the NMR spectrum results of medulla and cerebral cortex showed that, 21 potential metabolites were identified to participate in the mechanism of pathogenesis of GML and the regulation of electro-acupuncture, including 15 in medulla and 10 in cerebral cortex. Metabolic pathway analysis showed that the differential metabolites involved 19 metabolic pathways, which could be divided into energy, neurotransmitters, cells and cell membrane and antioxidation according to their functions. The correlation analysis of stomach, medulla and cerebral cortex shows that the stimulation signal of GML may reach the cerebral cortex from the stomach through medulla, and electro-acupuncture can treat GML by regulating the central nervous system (CNS).

**Conclusions:**

4 days electro-acupuncture treatment can significantly improve gastric mucosal injury, and the curative effect tends to be stable in 7 days treatment. Meanwhile, the pathogenesis of GML and the efficacy of electro-acupuncture involve metabolic pathways such as energy, neurotransmitters, cells and antioxidation, and electro-acupuncture can treat GML by regulating CNS.

**Supplementary Information:**

The online version contains supplementary material available at 10.1186/s13020-022-00593-9.

## Introduction

Gastric mucosal lesion (GML) that often manifests as edema, erosion, even hemorrhage and necrosis of mucosal tissues, is the initial pathological part of refractory gastric diseases, which leads to the imbalance between the defense of gastric mucosa and the invasion of gastrointestinal digestive fluid. Meanwhile, GML will cause metabolic abnormalities in the body affecting people’s quality of life seriously [1]. The inducement of GML is related to excessive mental tension, improper diet and taking drugs that stimulate excessive gastric acid secretion (reserpine, aspirin and nonsteroidal anti-inflammatory drugs), and it can also be used as complications of other diseases [2]. In particular, the excessive use of alcohol is the most likely cause of GML [3]. Nowadays, the modern medical treatment of GML mainly includes inhibiting gastric acid, protecting gastric mucosa and eradicating helicobacter pylori. These therapies can help improve symptoms, but they have disadvantages such as drug side effects and tolerance, and difficult acceptance by patients [4]. Therefore, there is a demand to seek out a safety and effective complementary and alternative therapy of treating and preventing GML.

Acupuncture is an integral part of traditional Chinese medicine (TCM), which has abundant clinical experience in the treatment of digestive system diseases [5]. Electro-acupuncture, an improved version of traditional acupuncture that usually stimulates acupuncture points by manual manipulations of needles, which increases stimulation as well as improves clinical effects by delivering electrical pulses to needles, gets more application in clinical practice currently [6]. Reports have shown that electro-acupuncture had a good preventive and therapeutic effect on GML by promoting cell proliferation and mucosal repair [7, 8]. A randomized controlled trial (RCT) has shown that acupuncture can effectively alleviate chronic atrophic gastritis and improve the pathological state of gastric mucosa, and has good long-term curative effect [9].These studies show that acupuncture and moxibustion can improve GML caused by gastritis or gastric ulcer, with low side effects, high patient acceptance and good long-term curative effect.

Nuclear magnetic resonance (NMR)-based metabolomics, which has extensively applied to exploring therapeutic mechanisms of TCM including electro-acupuncture, can analyze quantities of samples during an extremely short time with the assistance of an auto sample instrument, moreover, can identify metabolic reactions and potential biomarkers [10]. Researchers found that the mechanism of electro-acupuncture in the treatment of GML may be related to brain-stomach interaction [11]. It contains physiological functions such as nerve, immunity and endocrine, and plays an important role in maintaining the integrity of gastric mucosa and improving GML [12]. The lesions of digestive system are closely related to the central nervous system (CNS), at present, it is considered that there may be upward or downward regulation between GML and CNS, mainly through vagus-adrenal axis, hypothalamic–pituitary–adrenal (HPA) axis and brain intestinal peptide. Increased studies have confirmed the interaction between stomach and CNS [13]. As a macro observation method, NMR-based metabonomics has advantages for observing the reinforcement between stomach and CNS, it can simultaneously and comprehensively observe the changes of metabolites in stomach and brain, and observe the brain stomach interaction of GML from a more wide perspective [14].

Our previous studies have proved that acupuncture at stomach meridian, especially Liangmen (ST 21) and Zusanli (ST 36), can treat GML effectively and regulate related metabolic pathways, and the curative effect and regulation are related to the treatment time [15, 16]. However, there are few studies on the central metabolic regulation mechanism of GML treated by electro-acupuncture at the meridian point of stomach meridian. In order to elucidate the overall effect of electro-acupuncture, ^1^H NMR technique was applied to studying the dynamic expression of cerebral cortex and medulla metabolites in GML rats and to exploring the central metabolic response mechanism of electro-acupuncture in promoting gastric mucosal injury repair.

## Methods and materials

### Chemicals and materials

0.25 mm × 25 mm acupuncture needle were purchased from Hanyi Medical Instruments Co., Ltd. (Beijing, China); neutral balsam, anhydrous ethanol, dimethylbenzene, methanol and sodium chloride were purchased from Wuhan Service Biotechnology Co., Ltd. (Wuhan, China); sodium 3-trimethylsilyl- (2,2,3,3-d4) -1-propionate(TSP), pentobarbital sodium salt and deuterium oxide (D_2_O) were purchased from Sigma-Aldrich, St, Louis, MI, USA); nitrogen (Xiamen Yidong Co., Ltd., Xiamen, China); CD3 Monoclonal Antibody and Ly-6G/Ly-6C Monoclonal Antibody were purchased from Invitrogen (Carlsbad, CA, USA); NF-κB p65 Antibody were purchased from Cell Signaling Technology company (Danvers, MA, USA); DAB (AR1022) and SABC (SA1020) chromogenic kit were purchased from Wuhan Boster Biological Technology (Wuhan, China); Hematoxylin solution and eosin solution for histological stain were purchased from Beijing Leagene Biotechnology Co., Ltd (Beijing, China). Unless otherwise specified, all other used chemicals were of analytical grade.

### Animals

54 Specified Pathogen Free(SPF) male Sprague Dawley (SD) rats, weighing 180 ± 20 g, were purchased from Wu's experimental animal center (Permit Number: SCXK160803004). All animals were fed in Xiamen university laboratory animal center where the feeding temperature was kept at 20–22 ℃ and the relative humidity was controlled at 65 ± 2% with 12 h light–dark cycle. In this study, the entire animal experimental protocol was approved by the Animal Ethics Committee of Xiamen University, and conformed to “Guide for the Care and Use of Laboratory Animals” issued by National Ministry of Science and Technology [17].

### Induction of GML rat model and experimental design

After adaptive feeding for 7d, all rats were initially divided into 3 groups (n = 18): Control group, GML model (GML) group and Electro-acupuncture (EA) group. According to the different intervention courses, rats in each group were randomly further divided into 3 subgroups (n = 6) as follows: EA treatment 1 day (T1) subgroup Control (T1) subgroup and GML (T1) subgroup for electro-acupuncture intervention course of 1 day; EA treatment 4 days (T4) subgroup Control (T4) subgroup and GML (T4) subgroup for electro-acupuncture intervention course of 4 days; EA treatment 7 days (T7) subgroup Control (T7) subgroup and GML (T7) subgroup for electro-acupuncture intervention course of 7 days. According to the method of Czekaj [18], except for the rats of Control group, all rats were established GML modeling by gavage with 75% ethanol solution (4 ml/kg body weight) after 24 h of fasting. The criterion of successful GML model is that ulcer focus can be seen clearly.

### Electro-acupuncture treatment

In this study, according to “The Veterinary Acupuncture of China” and Government Channel and Points Standard GB12346-90 of China, 2 acupoints in the stomach meridian of Foot-Yangming including Liangmen (ST21) and Zusanli (ST 36) were selected. After the GML modeling, the rats in the EA groups, fixed on the frame previously, were treated 30 min per day by electro-acupuncture at ST21 and ST36 for 1 day, 4 days and 7 days, while the rats in Control and GML groups were only fixed on the frame 30 min per day without any other operation. Besides, the output line of electro-acupuncture apparatus (Model G6805-2, Shanghai Medical Instruments High Tech Co., Ltd., Shanghai, China) were connected with ST36 (positive pole) and ST21 (negative pole) on the same side (intermittent wave: 4 Hz; irregular wave: 50 Hz) after the needle inserts 3 to 5 mm at acupoints.

### Sample collection

After electro-acupuncture treatment, the rats in 3 subgroups were sacrificed by anesthetized with 2% sodium pentobarbital solution in day 1, day 4 and day 7, respectively. Then, the stomach, cerebral cortex and medulla were collected. 1 × 1 cm gastric tissue was cut and fixed with 4% paraformaldehyde for histopathological examination. The cerebral cortex and medulla were taken out and put into the cryopreservation tube, snap-frozen in liquid nitrogen immediately and stored at − 80 ℃ for follow-up NMR metabonomic analysis.

### Histopathological examination of stomach

After the gastric tissue was fully fixed with 4% paraformaldehyde, it was regularly dehydrated, embedded in paraffin, paraffin sectioned, dewaxed and hydrated in xylene and gradient alcohol. Then, the tissues were stained by hematoxylin and eosin staining. After sealing, histopathological examination was performed under light microscope.

### Immunohistochemistry

The expressions of NF-κB p65, CD3 and Ly6G/Ly6C in gastric mucosa were detected by diaminobenzidine (DAB) labeled immunohistochemistry (IHC). The paraffin section of gastric tissue was obtained after fixation, dehydration, paraffin embedding and sectioning. After conventional dewaxing and gradient ethanol, antigen repair was carried out by microwave heating in 0.01 mol/L citric acid buffer; After inactivation of endogenous peroxide in 3% hydrogen peroxide (H_2_O_2_), it was blocked by 5% bovine serum albumin (BSA), incubated with corresponding primary antibody, secondary antibody and strain avidin biotin complex (SABC); Color rendering was carried out according to the instructions of DAB chromogenic kit. After sealing with neutral gum, the positive expression of NF-κB p65, CD3 or Ly6G/Ly6C was observed by optical microscope, and 3 visual fields were randomly selected in each section. The average optical density (AOD) was calculated by Image Pro Plus 6.0 (media cybernetics, USA).

### Samples preparation and ^1^H NMR experiments

The changes of metabolites in cerebral cortex and medulla oblongata were observed by ^1^HNMR-based metabonomics. cerebral cortex and medulla tissue were weighed 200 to 210 mg on the ice, samples put into the mixture solution of 600 ml methanol and 300 ml H_2_O, and then fully homogenize and vortex for 1 min. After ice bath for 10 min, 300 ml chloroform was added, vortex and ultrasonic (100 Hz, 5 min) was applied to fully mix. After centrifugation (13,000 rpm, 4 ℃, 15 min), the supernatant was taken and dried on a nitrogen blower, and then mixed with 600ul of D_2_O containing sodium 3-trimethylsilyl—(2,2,3,3-d 4)—1-propionate (TSP, 1 mm), 500ul was put into a 5 mm nuclear magnetic resonance tube and tested in Bruker 600 MHz spectrometer (Bruker AV600, Bruker Corporation, Switzerland). The Carr Purcell MeiboomGill (CPMG) pulse sequence was used in this study. The specific scanning parameters were set as follows: spectral width was 12.019 kHz, relaxation time was 320 ms, scanning times was 64, FID conversion, LB = 0.3 Hz, PW = 30 ℃ (12.7 μs), RD = 1.0 s. After the ^1^H NMR spectra were collected by Bruker NMR spectrometer, the metabolites in the ^1^H NMR spectra of all samples were identified by NMR metabolites database developed by our team, published literatures[] and chemical shift database of compounds (such as BMRB, http://www.bmrb.wisc.edu/Metabolomics/) and HMDB(http://www.hmdb.ca/).

### Multivariate data analyses

Signal denoising, phased and baseline corrections were processed on all spectra of cerebral cortex and medulla samples by MestReNova version 9.0.1 (Mestrelab Research, Santiago de Compostela, Spain). All spectra were peak aligned, the single peak reference value of TSP is set as 0.0 ppm, and the spectra were split at 0.01 intervals in the range of δ 0.5–9.0 ppm after removing the water peak in the range of δ 4.70–5.2 ppm. In order to compensate for concentration differences between samples, the standardization that integral values from each spectrum were normalized to the sum of all the integrals in the spectrum for further multivariate analysis was processing.

Spectral data was imported SIMCA-P version 14.1softwave (Umetrics, Sweden), Pareto-scaling (Par) was used to reduce noise and remove artifacts in model. The principal component analysis (PCA) under unsupervised pattern recognition was used to evaluate the intergroup separation by visual observation. In order to eliminate the impact of related factors on the grouping, clear up intra-group differences and strengthen differences among groups, the orthogonal partial least squares discriminant analysis (OPLS-DA) was used to obtain the corresponding S-plot for analysis of potential variables after evaluating the quality of OPLS-DA model by analyzing model goodness of fit (R2) and prediction ability (Q2). In the light of the variable importance in the project (VIP ≥ 1.00) and the independent sample t-test (*P* < 0.05), the endogenous differential metabolites were acquired.

### Correlation analysis of different metabolites between cerebral cortex, medulla and stomach

In order to analyze the relationship between gastric and central metabolites, we SPSS version 23.0 software (IBM Corporation, Armonk, NY, USA) was used to analyze the Pearson correlation between the gastric differential metabolites obtained in the previous study and these obtained in this study of cerebral cortex and medulla. *P* < 0.05 showed that there was a significant relationship between the two metabolites.

## Results

### Electro-acupuncture improves gastric mucosal destruction in GML rats

**Fig. 1:**
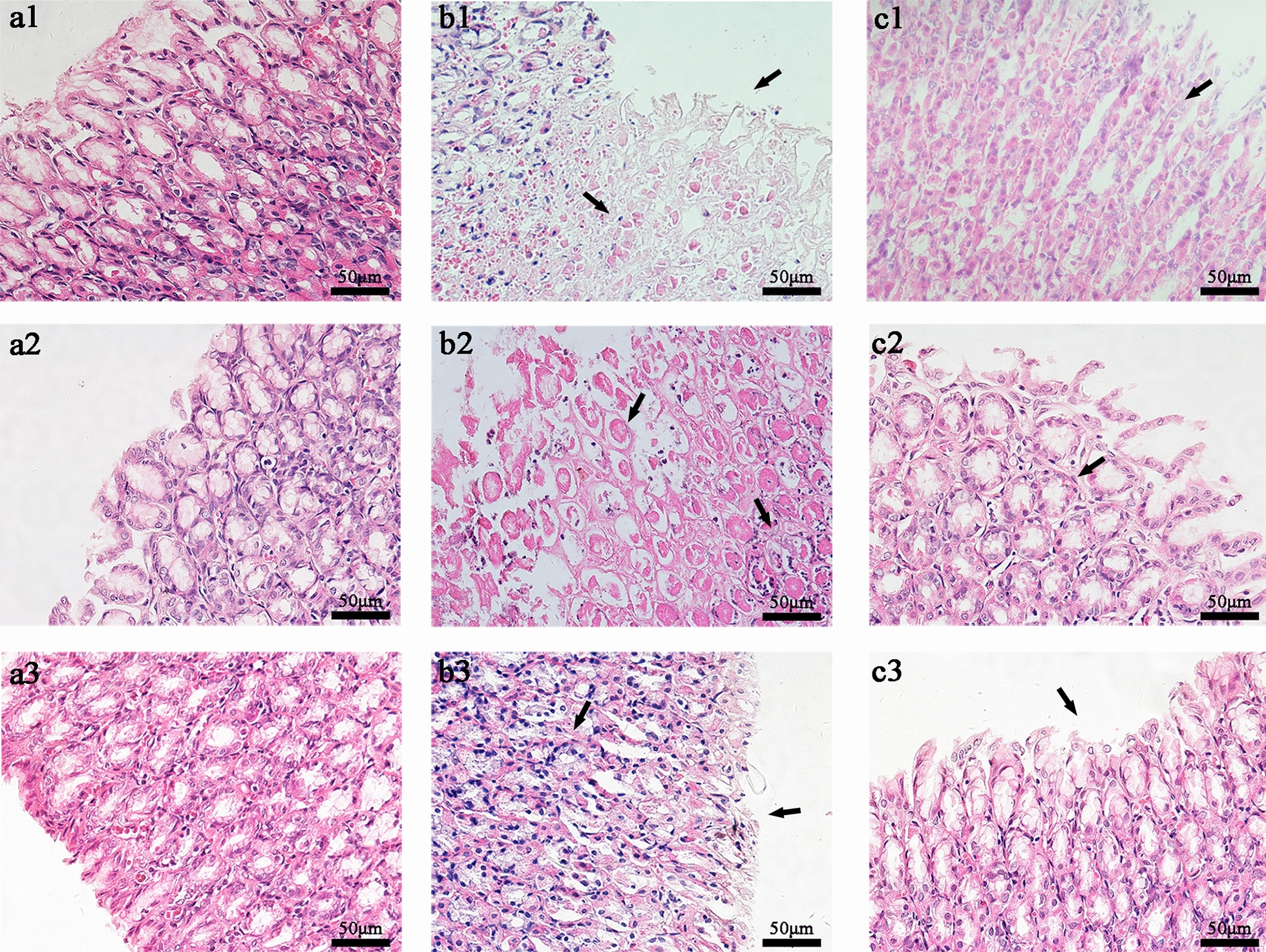
Histopathological observation of gastric mucosa in each group.** a1** gastric mucosa of rats in Control-T1 subgroup; **b1** gastric mucosa of rats in GML-T1 subgroup; **c1** gastric mucosa of rats in EA-T1 subgroup; **a2** gastric mucosa of rats in Control-T4 subgroup; **b2** gastric mucosa of rats in GML-T4 subgroup; **c2** gastric mucosa of rats in EA-T4 subgroup; **a3** gastric mucosa of rats in Control-T7 subgroup; **b3** gastric mucosa of rats in GML-T7 subgroup; **c3** gastric mucosa of rats in EA-T7 subgroup (magnification × 400; Scale bar, 50 µm)

The results of histological morphology of gastric tissues were shown in the Fig. 1 and Additional file 1: Fig. S1. In the Control groups, the epithelial of gastric mucosa were integrated, and the glands were normal in shape and orderly in arrangement. Meanwhile, there were clear boundaries between epithelial and ducts, the structures of mucosa and muscularis were integrated as well without inflammatory cells, hyperemia, edemas, ulcerative damages, atrophies, shedding or deformations. On the contrary, in the GML T1 group, the structure of gastric mucosal epithelial and glands arranged disorderly were severely damaged with a large number of necrotic and shedding cells, meanwhile a quantity of inflammatory cells and red blood cells could be observed. Compared with the GML T1 group, there were not notable ameliorations in the EA T1 group except that the hyperemia and the edema among tissues were slightly improved. The impaired gastric mucosal epithelial arranged disorderly, still existed in the GML T4 group, accompanied with inflammatory cells and red blood cells infiltrated as well as the hyperemia and the edema among tissues, while the structures of gastric mucosa epithelial and the glands in the EA T4 group were largely improved and clearer with a small amount of inflammatory cells and red blood cells infiltrated, furthermore, the glands arranged orderly and there were not distinct hyperemia and edema among tissue after 4 days electro-acupuncture treatment. What’s more, the gastric mucosa of rats in the GML T7 group were improved without obvious hyperemia and edema among tissues, compared with the previous, but there were still a small amount of mucosal epithelial structures destroyed. Remarkably, the histopathology examinations in the EA T7 group, including the gastric mucosa with integral structures and the glands arranged well without inflammatory cells and red blood cells infiltrated, were basically returned to normal.

**Fig. 2:**
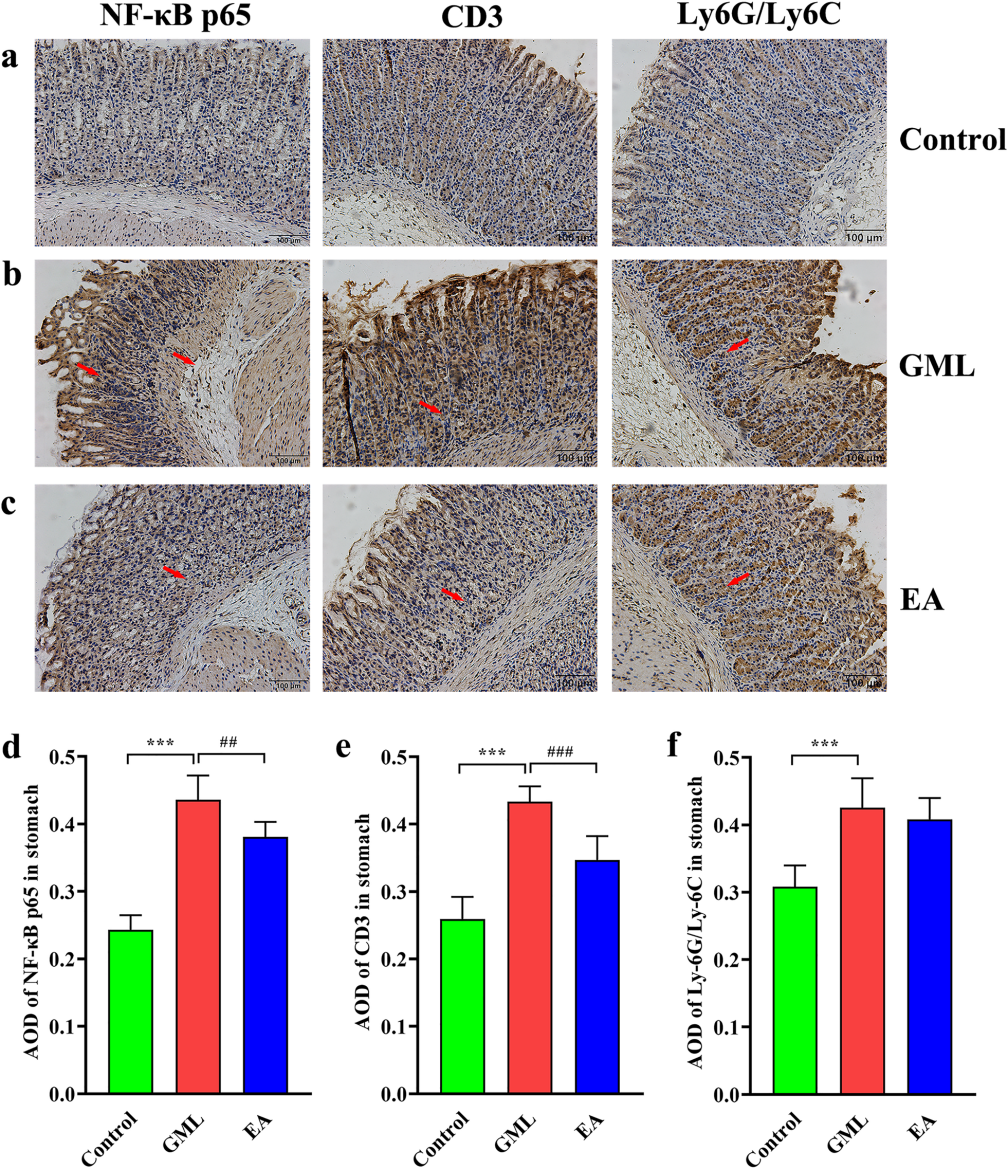
Observation of inflammation and immune cell expression in gastric mucosa in each group. **a** Representative photomicrographs of gastric NF-κB p65 immunohistochemical staining in each group. **b** Representative photomicrographs of gastric CD3 immunohistochemical staining in each group. **c** Representative photomicrographs of gastric Ly6G/Ly6C immunohistochemical staining in each group (magnification × 200; Scale bar, 100 µm). **d** AOD analysis of IHC results of NF-κB p65. **e** AOD analysis of IHC results of CD3.**f** AOD analysis of IHC results of Ly6G/Ly6C. ^***^P < 0.01 compared with the control group; ^###^*P* < 0.001, ^##^*P* < 0.01compared with the GML group

According to the results of HE staining of gastric mucosa, we found that 4 days of electro-acupuncture treatment (T4) is the key time point for the improvement of GML. Therefore, in order to evaluate the effect of electro-acupuncture on the degree of inflammation and immune cells of GML, we used IHC to detect expression of NF-κB p65, CD3 or Ly6G/Ly6C in gastric mucosa. As shown in Fig. 2, compared with the Control group, the positive expression of NF-κB p65, CD3 and Ly6G/Ly6C of gastric mucosa in GML group was more obvious, while it decreased to a certain extent after electro-acupuncture treatment. The results of AOD showed that NF-κB p65, CD3 and Ly6G/Ly6C in GML group were significantly higher than those in control group (all *p* < 0.001); The AOD of NF-κB p65 and CD3 in EA group was significantly lower than that in GML group (*p* = 0.003; *p* < 0.001), but there was no significant change in Ly6G/Ly6C (*p* = 0.416). This indicated that GML has obvious inflammation and immune cell infiltration compared with normal gastric mucosa, and 4-day electro-acupuncture can effectively inhibit gastric mucosal inflammation and CD3 + immune cell activation.

### Dynamic regulation of electro-acupuncture on differential metabolites in cerebral cortex and medulla

**Fig. 3:**
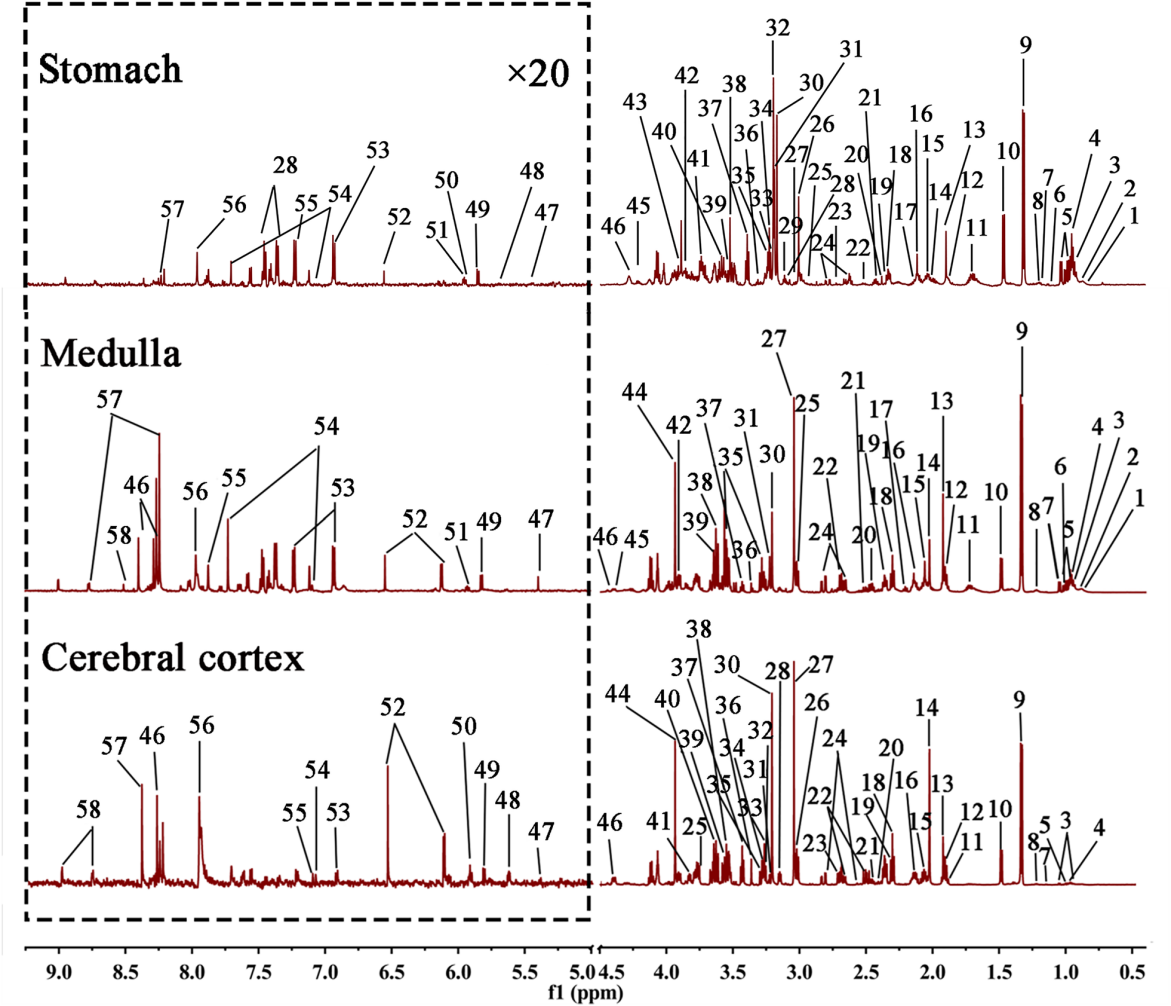
The typical ^1^H NMR spectra extracted from the tissues of stomach, medulla and cerebral cortex. (1, Low density lipoprotein (LDL); 2, Very low density lipoprotein (VLDL); 3, Isoleucine; 4, Leucine; 5, Valine; 6, 2-isoprene; 7, Ethanol; 8, Methylmalonic acid; 9, Lactate; 10, Alanine; 11, Lysine; 12, Gamma aminobutyric acid (GABA); 13, Acetate; 14, Acetyl aspartic acid; 15, Glutamate; 16, Glutamine; 17, Glutathione; 18, Pyruvic acid; 19, Oxaloacetate; 20, Succinate; 21, α-Ketoglutarate; 22, Citric acid; 23, Sarcosine; 24, Aspartate; 25, DMG; 26, Creatine; 27, Creatinine; 28, Phenylalanine; 29, Ethanolamine; 30, Choline; 31, Phosphocholine; 32, Glycerophosphocholine; 33, β-Glucose; 34, Taurine; 35, Myo-inositol; 36, Methanol; 37, α-Glucose; 38, Glycine; 39, Glycerol; 40, Glycogen; 41, Guanosine acetate; 42, Serine; 43, Hippurate; 44, Phosphocreatine; 45, Inosine; 46, Adenosine; 47, Allantoin; 48, Uridine 5'-diphosphoglucose; 49, Uracil; 50, Cytidine; 51, Uridine; 52, Fumarate; 53, Tyrosine; 54, 3-Methylhistidine; 55, Histidine; 56, Xanthine; 57, Nicotinamide; 58, Formate)

The typical ^1^H NMR spectra extracted from the tissues of cerebral cortex and medulla were displayed in Fig. 3, of which 56 endogenous metabolites were identified and the related chemical signals were displayed in Additional file 1: Tables S1–S3.

**Fig. 4:**
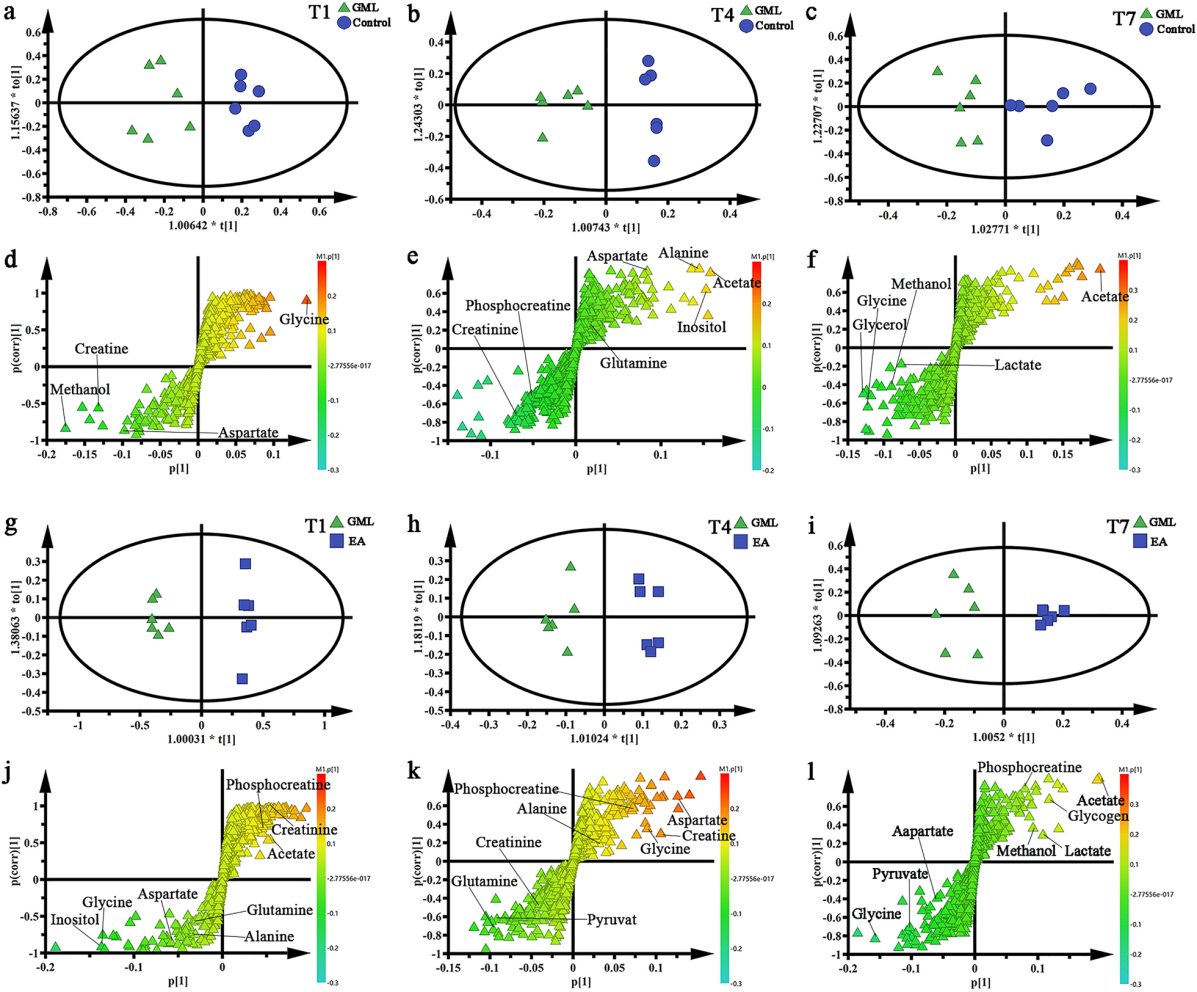
OPLS-DA scores plots and corresponding S-plots from medulla of rats. **a** and** d** scores plots and S-plots in Control-T1 and GML-T1 (R2X = 0.66cum, R2Y = 0.896cum, Q2 = 0.798cum); **b** and** e** scores plots and S-plots in Control-T4 and GML-T4 (R2X = 0.49cum, R2Y = 0.918cum, Q2 = 0.719cum); **c** and** f** scores plots and S-plots in Control-T7 and GML-T7 (R2X = 0.538cum, R2Y = 0.797cum, Q2 = 0.527cum); **g** and** j** scores plots and S-plots in GML-T1 and EA-T1 (R2X = 0.651cum, R2Y = 0.989cum, Q2 = 0.925cum); **h** and** k** scores plots and S-plots in GML-T4 and EA-T4 (R2X = 0.413cum, R2Y = 0.954cum, Q2 = 0.596cum); **i** and** l** scores plots and S-plots in GML-T7 and EA-T7 (R2X = 0.584cum, R2Y = 0.928cum, Q2 = 0.8cum)

**Fig. 5:**
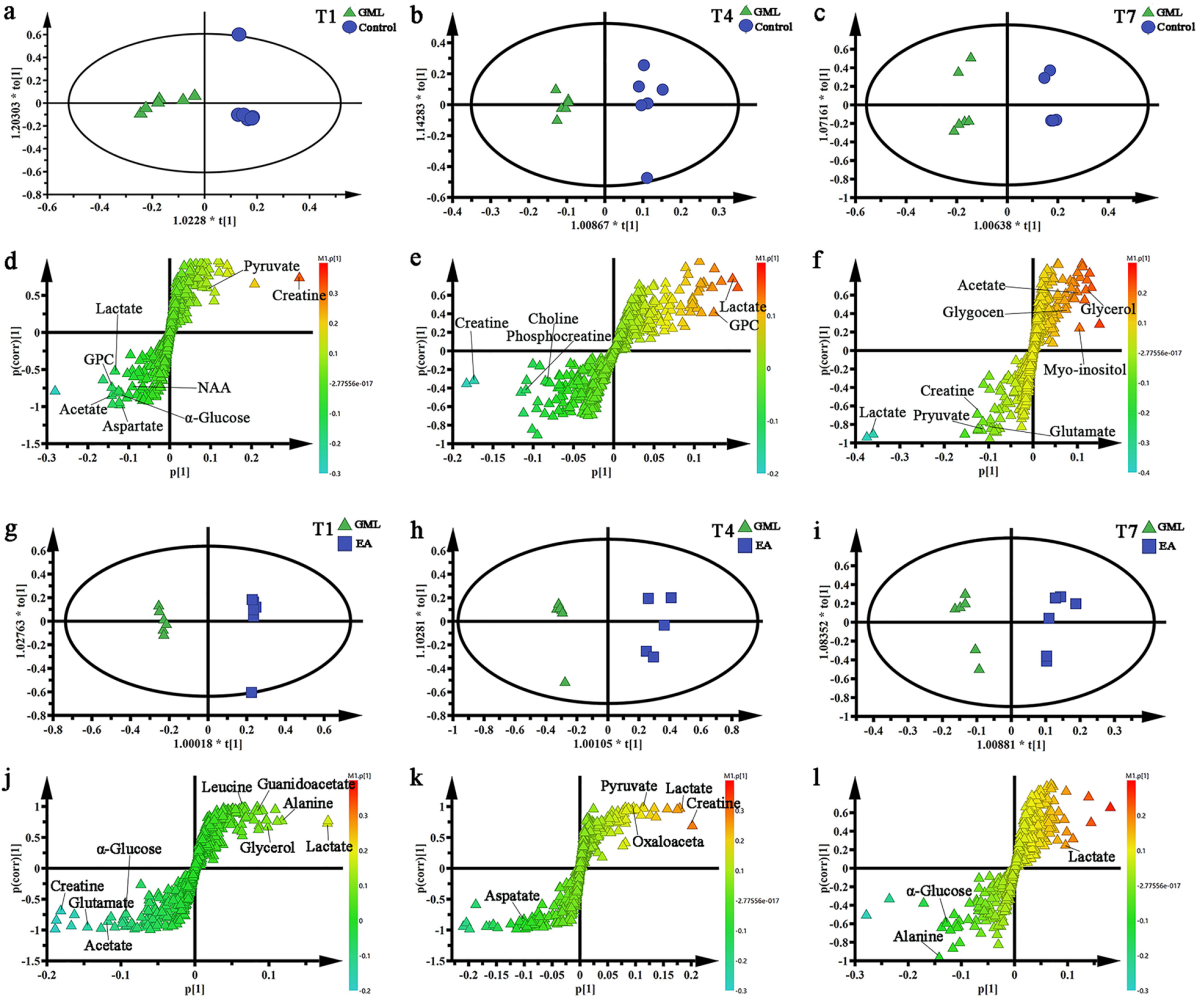
OPLS-DA scores plots and corresponding S-plots from cerebral cortex of rats. **a** and** d** scores plots and S-plots in Control-T1 and GML-T1 (R2X = 0.637cum, R2Y = 0.892cum, Q2 = 0.793cum); **b** and** e** scores plots and S-plots in Control-T4 and GML-T4 (R2X = 0.318cum, R2Y = 0.977cum, Q2 = 0.795cum); **c** and** f** scores plots and S-plots in Control-T7 and GML-T7 (R2X = 0.538cum, R2Y = 0.797cum, Q2 = 0.527cum); **g** and** j** scores plots and S-plots in GML-T1 and EA-T1 (R2X = 0.842cum, R2Y = 0.999cum, Q2 = 0.991cum); **h** and** k** scores plots and S-plots in GML-T4 and EA-T4 (R2X = 0.738cum, R2Y = 0.979cum, Q2 = 0.966cum); **i** and** l** scores plots and S-plots in GML-T7 and EA-T7 (R2X = 0.812cum, R2Y = 0.958cum, Q2 = 0.807cum)

Due to the complexity of the ^1^H NMR spectra, it’s difficult for visual inspection of ^1^H NMR spectra to distinguish differences of cerebral cortex and medulla tissues among groups, consequently, the OPLS-DA was used to discover any possible variables related to all groups. We found that in the medulla (Fig. 4a–c) and cerebral cortex (Fig. 5a–c), there was a clear separation between the Control groups and the GML groups, suggesting that there were significant metabolic changes in the GML rats. By the same way, as was demonstrated in Fig. 4g–i and Fig. 5g–i, the EA groups also separated obviously from the GML groups, indicating that electro-acupuncture treatment had a prominent effect on the GML rats at electro-acupuncture treatment 1 day, 4 days and 7 days, respectively. What’s more, the corresponding S-plot and t-test were applied to screening out the specific changes of metabolites related to medulla (Fig. 4d–f and j–l) and cerebral cortex (Fig. 5d–f and j–l) tissues among the N groups, the M groups and the A groups. There were following changes.

In medulla, the changes of differential metabolites of GML and the dynamic regulation of electro-acupuncture showed that, in T1 subgroup, compared with control group, the concentration of alanine, glutamate, glutamine, aspartate, taurine, α-glucose and pyruvate increased and leucine, glycine, serine, creatine, choline, inosine and phenylalanine decreased in GML group. After 1 day electro-acupuncture treatment, the expression of alanine, glutamine, aspartate, taurine, α-glucose and pyruvate were down-regulation, while glycine, serine, creatine, inosine and phenylalanine shown up-regulation. In T4 subgroup, GML can increase the content of serine, creatine, inosine and reduce the content of alanine, glutamine, aspartate, taurine, α-glucose. While, 4 days treatment could down-regulate the expression of glutamate, serine and pyruvate and up-regulate the content of aspartate, taurine and phenylalanine. In T7 subgroup, GML significantly increased glutamate and serine, while electro-acupuncture treatment for 7 days could reduce glycine, choline and phenylalanine, and increase the expression of α-glucose.

The changes of differential metabolites of GML and the dynamic regulation of electro-acupuncture in the cerebral cortex showed that, in T1 subgroup, compared with control group, the expression of leucine, acetate, pyruvate, aspartate, creatine and lactate increased, while γ-aminobutyric acid (GABA), succinate, α-ketoglutarate and glutamine reduced in GML group. In the EA group, after 1 day treatment, the concentration of acetate, pyruvate, creatine and glutamine were down-regulation, while succinate and α-ketoglutarate were up-regulation. Interestingly, the expression of leucine was further increased after treatment. In T4 subgroup, the content of GABA and glutamine reduced in GML rat, 4 days electro-acupuncture can help raise glutamine, besides, the content of pyruvate and lactate increased while aspartate and creatine decreased in EA group. In T7 subgroup, the concentration of GABA and acetate reduced, while, pyruvate and lactate ascended in GML group. However, their expressions have not changed significantly after treatment.

Additional file 1: Figs. S2–S7 would show the relative abundance of potential differential metabolites.

### Electro-acupuncture regulates GML based on metabolic network

**Fig. 6:**
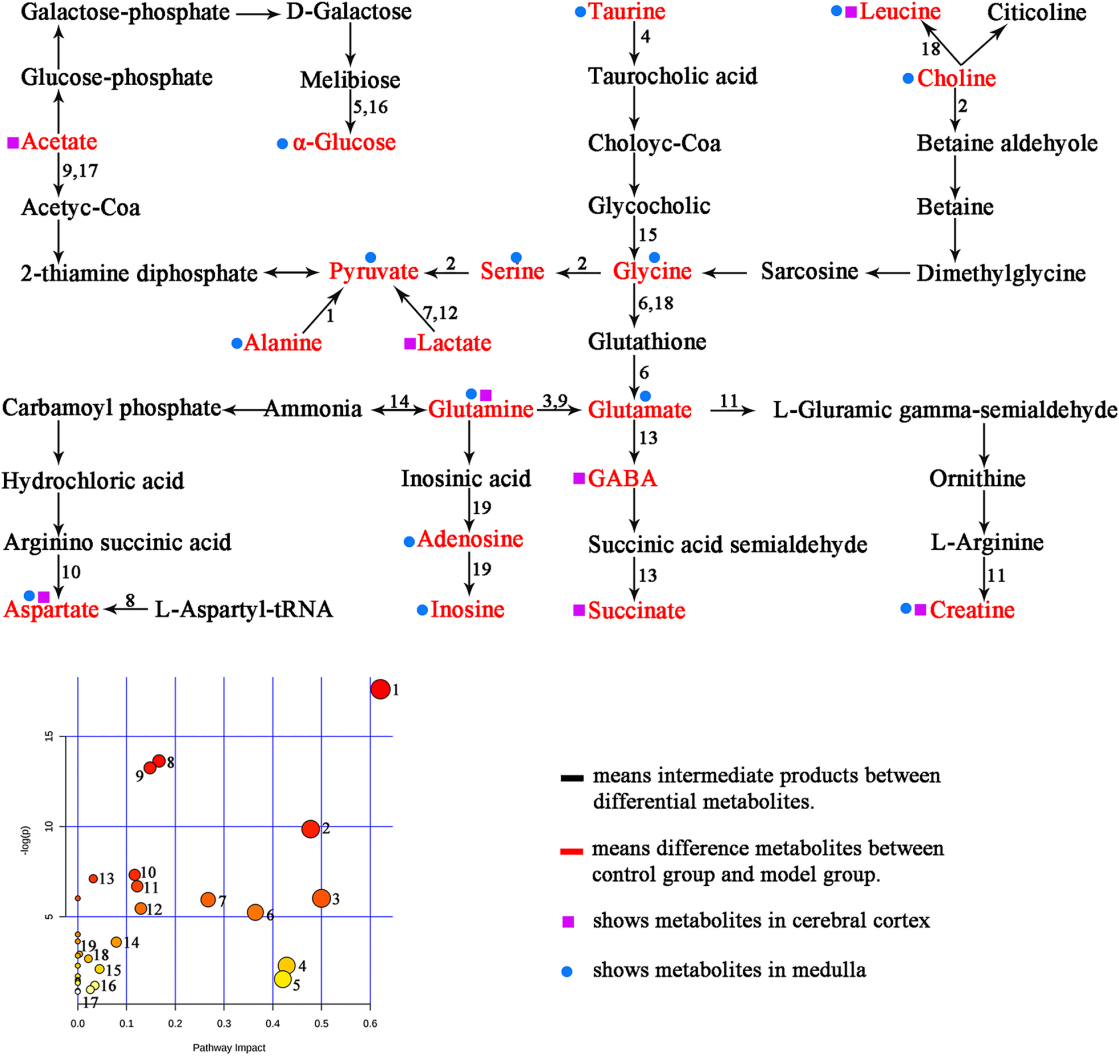
Metabolic pathways related to ^1^H NMR based differential metabolites in rats. (1, Alanine, aspartate and glutamate metabolism; 2, Glycine, serine and threonine metabolism; 3, D-Glutamine and D-glutamate metabolism; 4, Taurine and hypotaurine metabolism; 5, Starch and sucrose metabolism; 6, Glutathione metabolism; 7, Pyruvate metabolism; 8, Aminoacyl biosynthesis; 9, Glyoxylate and dicarboxylate metabolism; 10, Arginine biosynthesis; 11, Arginine and proline metabolism; 12, Glycolysis/Gluconeogenesis; 13, Methyl butyrate metabolism; 14, Citrate acid cycle (TCA cycle); 15, Primary bile acid biosynthesis; 16, Galactose metabolism; 17, Glycerophospholipid metabolism; 18, Cysteine and methionine metabolism; 19, Purine metabolism). The purple square represents different metabolites in cerebral cortex; the blue circle represents different metabolites in medulla. Black font means intermediate products between differential metabolites; red means difference metabolites between control and GML group

By analyzing the NMR metabolomic spectrum of cerebral cortex and medulla, 21 differential metabolites were obtained, including 15 in medulla (taurine, leucine, choline, α-glucose, pyruvate, serine, glycine, alanine, glutamine, glutamate, adenosine, aspartate, inosine, phenylalanine and creatine) and 10 in cerebral cortex (leucine, GABA, acetate, pyruvate, aspartate, creatine, lactate, succinate, α-ketoglutarate and glutamine). After analyzing the related metabolic pathways of these differential metabolites by MetaboAnalyst 5.0 tool (https://www.metaboanalyst.ca/), it was found that these differential metabolites were mainly related to 19 metabolic pathways and could form a network of metabolic pathways (Fig. 6), which could be further divided into 4 main metabolic pathway according to their physiological function, including 1.Energy (ATP) related metabolism, 2. Neurotransmitter related metabolism, 3. Cell and cell membrane related metabolism, 4. Antioxidant related metabolism (Fig. 6).

### The relationship between gastric-CNS metabolites in GML and the effect of Electro-acupuncture

**Fig. 7:**
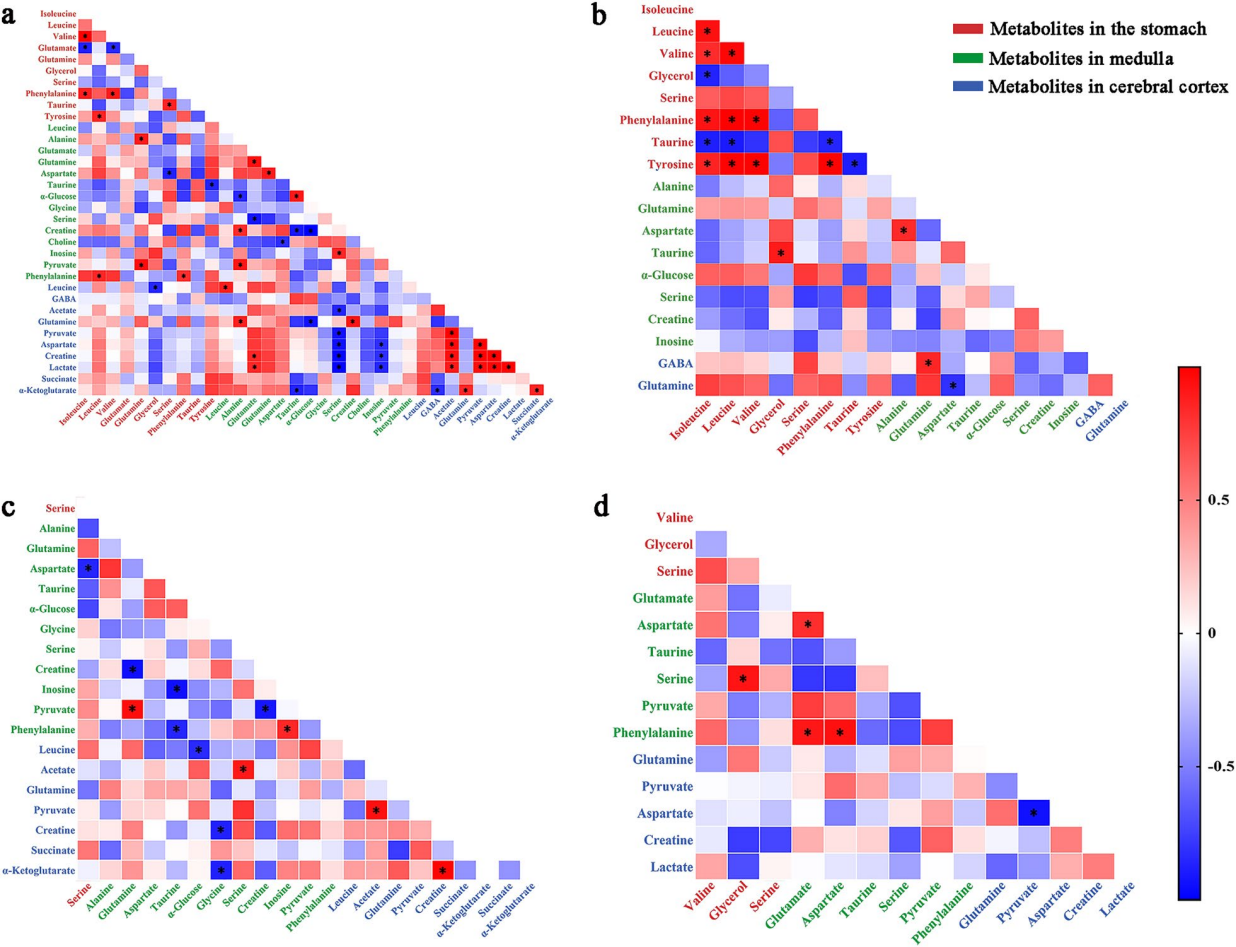
Correlation analysis of potential metabolites in stomach, medulla and cerebral cortex of rat.** a** correlation analysis of differential metabolites in GML-T1 subgroup; **b** correlation analysis of differential metabolites in GML-T4 subgroup; **c** correlation analysis of differential metabolites in EA-T1 subgroup; **d** correlation analysis of differential metabolites in EA-T4 subgroup. The red font represents different metabolites in gastric tissue; the green font represents different metabolites in medulla; the blue font represents different metabolites in cerebral cortex. The red area represents a positive correlation between the two metabolites, and the blue area represents a negative correlation

Previous studies [15] of our team showed that, there were differences in the expression of isoleucine, leucine, valine, glutamate, glutamine, glycerol, serine, phenylalanine, taurine and tyrosine in GML. In order to explore the relationship between the different metabolites of CNS (medulla and cerebral cortex) and stomach in GML and the regulatory effect of electro-acupuncture on them, we carried out Pearson correlation analysis on different metabolites in stomach, medulla and cerebral cortex. The results show that, the correlation of different metabolites in the same tissue is obvious. In order to observe the correlation of potential metabolites in different tissues, we only focus on the correlation changes of potential metabolites between different tissues. The specific results are as follows (Fig. 7).

1. GML-T1 subgroup. ***a.*** In gastric tissue, leucine was positively correlated with phenylalanine in medulla (*r* = 0.848, *p* = 0.033), glutamine was positively correlated with alanine and pyruvate in medulla (*r* = 0.89, *p* = 0.017; *r* = 0.941, *p* = 0.005), glycerol was negatively correlated with leucine in cerebral cortex (*r* = − 0.852, *p* = 0.031), serine was negatively correlated with aspartate in medulla (*r* = − 0.874, *p* = 0.023), phenylalanine was positive correlation in gastric tissue and medulla (*r* = 0.816, *p* = 0.048), tyrosine was negatively correlated with taurine in medulla (*r* = − 0.868, *p* = 0.025). ***b.*** The leucine was positively correlation in medulla and cerebral cortex (*r* = 0.88, *p* = 0.021); in medulla, alanine was positively correlated with glutamine in cerebral cortex (*r* = 0.874, *p* = 0.023), glutamate was positively correlated with creatine and lactate in cerebral cortex (*r* = 0.847, *p* = 0.033; *r* = 0.845, *p* = 0.034), and taurine was positively correlated with α-Ketoglutarate in cerebral cortex (*r* = − 0.864, *p* = 0.026), α-Glucose was negatively correlated with glutamine in cerebral cortex (*r* = − 0.927, *p* = 0.008), serine was negatively correlated with acetate, pyruvate, aspartate, creatine and lactate in cerebral cortex (*r* = − 0.861, *p* = 0.028; *r* = − 0.931, *p* = 0.007; *r* = − 0.94, *p* = 0.005; *r* = − 0.947, *p* = 0.001; *r* = − 0.973, *p* = 0.001), creatine was positively correlated with glutamine in cerebral cortex (*r* = 0.919, *p* = 0.01), and inosine was negatively correlated with aspartate, creatine and lactate in cerebral cortex (*r* = − 0.819, *p* = 0.046; *r* = 0.818, *p* = 0.047; *r* = 0.854, *p* = 0.031) (Fig. 7a).
2. GML-T4 subgroup. In gastric tissue, glycerol was positively correlated with taurine in medulla (*r* = 0.89, *p* = 0.018). In medulla, glutamine was positively correlated with GABA in cerebral cortex (*r* = 0.847, *p* = 0.031), and aspartate was negatively correlated with glutamine (*r* = − 0.814, *p* = 0.049) (Fig. 7b).
3. EA-T1 subgroup. In gastric tissue, serine was negatively correlated with aspartate in medulla (*r* = − 0.853, *p* = 0.031). In the medulla, α-Glucose is negatively correlated with leucine in cerebral cortex (*r* = − 0.836, *p* = 0.038), and glycine is negatively correlated with creatine and α-ketoglutarate in cerebral cortex (*r* = − 0.873, *p* = 0.023; *r* = − 0.874, *p* = 0.023), serine was positively correlated with acetate in cerebral cortex (*r* = 0.9, *p* = 0.014) (Fig. 7c).

EA-T4 subgroup. The glycorol in gastric tissue was positively correlated with serine in medulla (*r* = 0.917, *p* = 0.01) (Fig. 7d).

## Discussion

In this study, the ethanol induced GML rat model was established to study the effect of different electro-acupuncture treatment period on GML and the changes of metabolites in CNS. The histopathological results of gastric mucosa showed that, GML had obvious gastric mucosal ulcer, gland destruction and inflammatory cell infiltration on day 1 and 4, showing a typical pathological state of gastric mucosal injury. On day 7, the gastric mucosal injury was slightly improved. Electro-acupuncture treatment for 1 day could not significantly improve the state of GML, importantly, 4 days of treatment could effectively improve the symptoms of gastric mucosa, and the curative effect tended to be stable on the 7th day. The NMR-based metabolome technology shown that, 21 potential metabolites in medulla and cerebral cortex were found in GML, including 15 in medulla and 11 in cerebral cortex. The metabolic network analysis of potential metabolites found that a total of 19 metabolic pathways were involved, which were related to them. According to their functions, they were divided into 4 categories: energy (ATP) related metabolism, neurotransmitter related metabolism, cell and cell membrane related metabolism, antioxidant related metabolism. There is also a correlation between the different metabolites of the stomach, medulla and cerebral cortex. Therefore, this study shows that acupuncture can effectively improve GML and regulate the related metabolites of CNS, and the changes of these metabolites are related to these of the stomach.

GML is common in gastric ulcer and gastritis. The increasing evidences shown that electro-acupuncture has a positive effect on GML in gastric ulcer and gastritis. Li et al. [19] showed that electro-acupuncture can effectively inhibit gastric mucosal injury caused by gastric ulcer, the curative effect is similar to omeprazole, and the mechanism may be related to the regulation of Toll-like receptors 4/Nuclear factor kappa-B (TLR4/NF-κB) pathway. A clinical research shows that acupuncture can effectively improve the symptoms of patients with chronic atrophic gastritis and protect gastric mucosa [9]. This is consistent with the results of this study. Our previous studies also showed that electro-acupuncture can repair gastric mucosal glands and reduce inflammatory infiltration [7, 12, 14–16, 20–22]. The above studies confirmed that electro-acupuncture can protect gastric mucosa and reduce the symptoms of GML.

Gastric mucosal is a natural barrier to protect the integrity of gastric wall, the lesion of gastric mucosal may cause metabolic disorders in amino acids, sugars and lipids. Moreover, according to related literature, such kinds of metabolic disorders are key pathological manifestations of gastric mucosal injury [23]. Each metabolite with concentration changes in the process of inflammatory reactions may become a potential candidate for regulating the activity of inflammatory factor, because metabolites have been proved to be tightly regulated in the process of inflammatory reactions recently, and there is a clear link between inflammatory reactions and metabolic disorders [24, 25]. Previous literatures have illustrated that electro-acupuncture had a significant effect on gastric mucosal lesion while the mechanism haven’t been revealed yet, which was consistent with the theory of TCM that acupuncture at acupoints of stomach meridian, especially Liangmen (ST 21) and Zusanli (ST 36), can harmonize the qi of stomach and relieve pain. The relationship between GML and CNS has attracted increased attention in recent years. Gyires [26] reviewed the relationship between the integrity of gastric mucosa and the CNS, indicating that the CNS is involved in the regulation of gastrointestinal functions, including the vagus nerve dependent way of hypothalamus and dorsal vascular complex (DVC) receptor, Glutamate participates in the synaptic links of nucleus tractus solidarius (NTS) and dorsal motor nucleus of the vague (DMNV), and amino acids, such as N-methyl-D-aspartic acid (NMDA), play a central role in the protection of gastric mucosa. A breakthrough study [27] revealed from the perspective of neuroanatomy that electro-acupuncture can activate PROKR2^ADV^ neurons in dorsal root ganglion (DRG) through distal acupoint ST36, upward to NTS-DMNV, down through vagus nerve to induce the release of anti-inflammatory factors and inhibit peripheral inflammation. Our previous study also shows [14] that the metabolic pathway of amino acids plays an important role in electro-acupuncture in the treatment of chronic atrophic gastritis (CAG). Cerebral cortex and medulla, as advanced nerve centers, involved in the regulation of visceral movements and numerous glandular secretions, where the concentration changes of metabolites are closely related to the therapeutic mechanism of electro-acupuncture [28]. Based on our previous studies, it was shown that mucosal repair after gastric mucosal injury treated by electro-acupuncture was mainly regulated by different metabolites and multiple metabolic pathways, and we classify metabolic pathways into the following categories.

## Energy metabolism

Creatine is generated by phosphocreatine, as an important carrier for energy storage and transport, and it released adenosine triphosphate (ATP) to supplement energy, maintain normal physiological activities, and has neuroprotective function. At the same time, creation can also promote the recycling of ATP, mainly through adenosine diphosphate (ADP) provides phosphate groups and converts them into ATP to promote the recovery of ATP. Acetate can drive the increase of acetyl-CoA metabolism [29, 30]. Acetyl-CoA is the premise of extensive activity of many organisms. It is the energy supply of mitochondria and the core of glycerol phosphatide, glycoxylate and dicarboxylate metabolism [31]. In this study, the content of creatine in GML-T1 and T4 groups in cerebral cortex showed an upward trend continuously, indicating that the cell membrane was destroyed, more energy was released through its own regulatory system for self-repair of the cell membrane, while the levels of creatine and acetate in EA group were stable after the 4 days treatment, indicating that electro-acupuncture can play a certain role in protecting and repairing the gastric mucosa. Glucose is the main energy material in the body, which can provide energy for cells. Glycerol is a three carbon material, which forms the skeleton of fatty acids in fat and can be converted into glucose [32]. In this study, the content of α-glucose in EA group in medulla increased gradually, indicating that electro-acupuncture can promote the body to release energy to maintain the energy supply required by the body to repair gastric mucosa to a certain extent, while the content ofα-glucose in GML group always showed a downward trend, suggesting that the body may not start to repair cell membrane. Alanine is an important participant and regulator in glucose metabolism, and like glycine, it is an inhibitory neurotransmitter in the brain [33]. In medulla, the content of T1 subgroup changed, indicating metabolic disorder. After 4 days of electro-acupuncture treatment, the content of alanine in EA-T4 group began to recover, and the content of alanine in EA-T7 group reached the normal level. It shows that electro-acupuncture intervention can prevent and treat gastric mucosal injury by regulating metabolism to a certain extent. Inosine and adenosine are mainly involved in purine metabolism. Inosine is a purine nucleoside, an intermediate product of the degradation of purine and purine nucleosides (such as hypoxanthine), and also exists in the anticodon of some transfer RNA molecules. Adenosine is transformed into inosine through RNA editing, which becomes a neural transmission in the brain, and plays a vital role in energy conversion like ATP and ADP [34, 35]. In medulla, adenosine content in T1 subgroup changed, while adenosine in EA-T4 group reversed and increased to near normal level, and adenosine content in EA-T1 group showed an upward trend. After 4 days of electro-acupuncture treatment, the content of adenosine in EA-T4 group was close to normal value and tended to be stable, indicating that electro-acupuncture needed to consume certain energy in repairing gastric mucosal injury.

### Neurotransmitter metabolism

Glutamine is the precursor of glutamate neurotransmitter and the most abundant rapid excitatory neurotransmitter in the nervous system. At the same time, it is also involved in maintaining the integrity of gastrointestinal mucosa [36, 37]. In this study, glutamine in cerebral cortex showed a downward trend in T1 subgroup, indicating that the cell membrane had been seriously damaged, while glutamine in EA-T4 group had been reversed, and there was no significant difference in T3 subgroup. In the medulla, the contents of glutamate and glutamine also changed accordingly, the contents of them in EA group were always close to the level of Control group, the contents of them in GML-T1 and T4 groups were always higher than those in Control and EA group, while these in GML-T7 group showed a downward trend, indicating that the damage of gastric mucosa had improved after the 4 days of electro-acupuncture which had played a role in protecting gastric mucosa. Aspartate was found to stimulate neurons and promote the release of neurotransmitters in the 1960s, while, recent researchs have confirmed that excessive aspartate can also directly damage neurons [38]. In this study, the content of aspartate in medulla changed significantly, and the content in T1 subgroup increased, indicating that neuronal cells were inhibited, while the content of aspartate in EA-T4 group had reversed and returned to the normal level, the content in EA-T7 group showed a stable trend, and the content of aspartate in GML-T7 group also began to reverse, indicating that electro-acupuncture could significantly inhibit the release of excitatory neurotransmitters and promote the release of inhibitory neurotransmitters, which shows that electro-acupuncture can protect the integrity of gastric mucosa to a certain extent. The content of aspartate basically returned to normal after 7 days, while GML-T7 group also relied on its own regulatory system to repair itself.

### Cell and cell membrane metabolism

There are two possible transformations of choline: oxidation to betaine or synthesis of phosphotidylcholine [39]. Importantly, a phosphatidylcholine in gastric mucosa can resist gastric injury caused by non-steroidal anti-inflammatory drugs (NSAIDs) [40]. In this study, the choline content of medulla changed, and the content of choline in T1 subgroup decreased significantly, indicating that the gastric mucosa was seriously damaged and the cytoskeleton was decimated. After 4 days of treatment, the content of choline in EA-T4 group returned to near normal level, indicating that electro-acupuncture played a role in preventing and controlling the damage of gastric mucosa to a certain extent. Succinate is the product of methylbutyrate metabolism and tricarboxylic acid (TCA) cycle, at the same time, it also participates in the synthesis of ATP and provides energy for cells [41]. In the cerebral cortex, the content of succeed in EA-T1 and T4 groups increased, indicating that after the cell membrane was damaged, it shows that after the cell membrane is destroyed, it needs to consume a lot of energy to promote the recovery of cell membrane by treatment, and the content of succinate in EA-T7 group decreased, indicating that the cell membrane has recovered to a certain extent and does not need a lot of energy to repair. Leucine is a branched chain amino acid (BCAAs), which mainly participates in cell-related metabolism by promoting mitochondrial metabolism, and finally maintains gastrointestinal homeostasis and improves immune related functions [42, 43]. In the cerebral cortex, the content of leucine in EA-T1 and T4 groups showed an upward trend and was always higher than that in GML-T1 and T4 groups. The content of leucine in EA-T7 group tended to be stable, and the content in GML-T7 group began to increase, indicating that treatment began to consume a lot of energy to repair gastric mucosa and reached stability in 7 days treatments. In medulla, the content of leucine in EA group was always higher than that in GML group, which also showed that electro-acupuncture was always in the stage of cell membrane repair.

### Antioxidant metabolism

Glycine is not only an antioxidant, but also a precursor of a variety of proteins which will rise significantly when the body encounters severe stress, and can enhance the antioxidant capacity of body tissues [44, 45]. The serine is the products of glycine after glycine, serine and threonine metabolism pathway. Serine is the precursor of a variety of amino acids, which not only helps to produce immunoglobulin and antibody, but also enhances the antioxidant response of the body, and plays a very important role in the prevention and treatment of diseases [46]. In the medulla, the content changes of glycine and serine in T1 subgroup showed a synchronous state. After that, the content of glycine and serine in GML-T4 and T7 groups increased continuously, indicating that the cell membrane was damaged. After 7 days of electro-acupuncture treatment, the content of glycine and serine in EA-T7 group was close to the normal level, indicating that 7 days of treatment played a certain role in the repair of cell membrane. Taurine is an antioxidant, which has the functions of maintaining cell membrane stability, regulating cell osmotic pressure and regulating cell signal transmission [47]. In medulla, the content of taurine in T1 subgroup decreased, indicating that the cell membrane structure was significantly damaged. After electro-acupuncture, the content of taurine in EA-T4 and T7 groups increased, indicating that electro-acupuncture intervention can maintain the stability of cell membrane by promoting the release of taurine to a certain extent, so as to prevent and treat gastric mucosal injury.

The relationship between peripheral inflammation such as gastrointestinal inflammation and CNS has attracted growing attention. Several recent breakthrough studies have shown that CNS can regulate the changes of peripheral inflammation through a variety of ways. Liu's research [27] shows that, when electro-acupuncture stimulates acupoints, it will activate the fibers of PROKR2 neurons of nervus peroneus communis, and the stimulation signals will be transmitted successively through DRG in limb level of spinal cord (L4-L5), NTS and dorsal motor nucleus of DMNV, so as to regulate the vagus-adrenal pathway and regulate inflammation. Carloni's research [48] has confirmed that gastrointestinal inflammation will lead to the destruction of digestive tract barrier, and lead to the dysfunction of brain barrier through wingless-type/catenin-beta 1 (Wnt/β-catenin) signal pathway, resulting in the abnormality of central anti-inflammatory function. The above studies show that the lesions of digestive system are closely related to CNS. Exploring the communication between them is of great significance to promote the pathogenesis of GML and the therapeutic mechanism of acupuncture. In this study, we analyzed the correlation between different metabolites in the stomach, medulla and cerebral cortex. It was found that on the first day of GML model preparation, the metabolites in the stomach were mainly related to the appearance of medulla, and there were more metabolite correlations between medulla and cerebral cortex, which may indicate that in the early stage of GML, the metabolic changes of the body affected the cerebral cortex from the stomach through medulla. Electro-acupuncture seems to be less involved in the connection between the stomach and CNS, but more involved in the connection between the medulla and the cerebral cortex. In general, from the perspective of metabolic changes, there is a relationship between stomach and CNS in GML. Electro-acupuncture can participate in the development of GML by regulating CNS.

## Conclusion

By analyzing the NMR spectra of the cerebral cortex and medulla of rats with GML combining with the histopathological evaluation, this study demonstrated that electro-acupuncture at ST 36 and ST 21 could effectively treat GML, which might be achieved by affecting the energy metabolism, neurotransmitter metabolism, cell and cell membrane metabolism, antioxidant metabolism and other related metabolic pathways in the CNS of rats, so as to prevent and protect cell membrane damage. After electro-acupuncture treatment for 1 day, the repair effect of gastric mucosa was not obvious, however, after 4 days of electro-acupuncture treatment, a reversal phenomenon appeared in most of metabolites contents, to a certain extent, and after 7 days of treatment, the call-back effect of the contents of metabolites tended to be stable. These elucidated that electro-acupuncture treatment should last at least 4 days and more for the treatment of GML. Considering the interaction of potential self-regulatory mechanisms in rats, the link between fluctuations in concentration and intervention time could not be visually displayed, but such changes of metabolites contents were still worthwhile to explore further. In this paper, the influence of the GML repair after electro-acupuncture treatment in the CNS was revealed, which laid a good foundation for the complete interpretation of the therapeutic effect of electro-acupuncture on GML.

## Supplementary Information

**Additional file 1.** Additional figures and tables.

## Data Availability

The datasets used and analysed during the current study are available from the corresponding author on reasonable request.

## Abbreviations

GML: Gastric mucosal lesions
EA: Electro-acupuncture
CNS: Central nervous system
TCM: Traditional Chinese medicine
NMR: Nuclear magnetic resonance
HPA: Hypothalamic–pituitary–adrenal
TSP: Sodium 3-trimethylsilyl-(2,2,3,3-d4)-1-propionate
T1: 1 Day treatment
T4: 4 Days treatment
T7: 7 Days treatment
PCA: Principal component analysis
OPLS-DA: Orthogonal partial least squares discriminant analysis
LDL: Low density lipoprotein
VLDL: Very low density lipoprotein
GABA: Gamma aminobutyric acid
TCA: Tricarboxylic acid
ATP: Adenosine triphosphate
ADP: Adenosine diphosphate
BCAAS: Branched chain amino acid
NTS: Nucleus tractus solitaries
DMNV: Dorsal motor nucleus of vagus
