# Supplementary material for: NMR-based metabonomics reveals the dynamic effect of electro-acupuncture on central nervous system in gastric mucosal lesions (GML) rats
Source: Chin Med. 2022 Mar 21;17:37. doi: 10.1186/s13020-022-00593-9 (PMC8935774; doi:10.1186/s13020-022-00593-9)
Supplement: Supplementary file 1 — Additional file 1. Additional figures and tables. [file 13020_2022_593_MOESM1_ESM.docx]

**NMR-Based Metabonomics Reveals the Dynamic Effect of Electro-acupuncture on Central Nervous System in Gastric Mucosal Lesions (GML) Rats**

Miaosen Huang^1†^, Yiwei Peng^2†^, Qida He^3†^, Linyu Lian^2^, Yichen Wang^2^, Longbin Zhang^1^, Yuan Zhang^4^, Jiacheng Shen^5*^, Zongbao Yang^1*^

^*^Correspondence: yangzb@xmu.edu.cn; shenjiacheng369@163.com

^†^ Miaosen Huang, Yiwei Peng and Qida He contribute equally to this work

^1^School of Medicine, Xiamen University, Xiamen, 361102, Fujian, China

^5^Shanghai University of Traditional Chinese Medicine, Shanghai 201203, Shanghai, China

Full list of author information is available at the end of the article


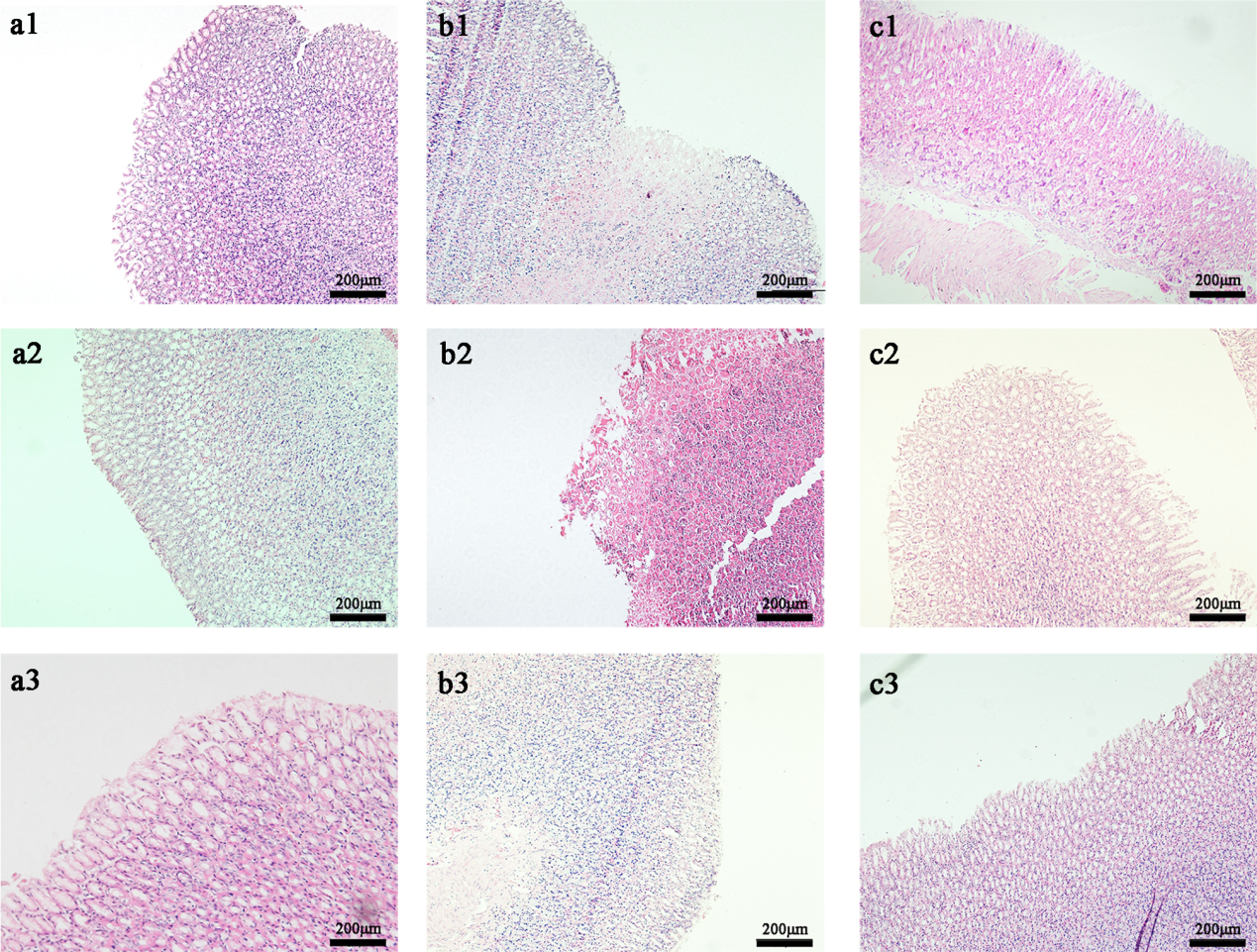


**Fig. S1** Histological examination of gastric mucosa from all groups.(a1, b1 and c1, rats in Control, GML and EA group at 1 day treatment; a2,b2 and c2, rats in Control, GML and EA group at 4 days treatment; a3, b3 and c3, rats in Control, GML and EA group at 7 days treatment;). Scale bars represent 200μm in each group.


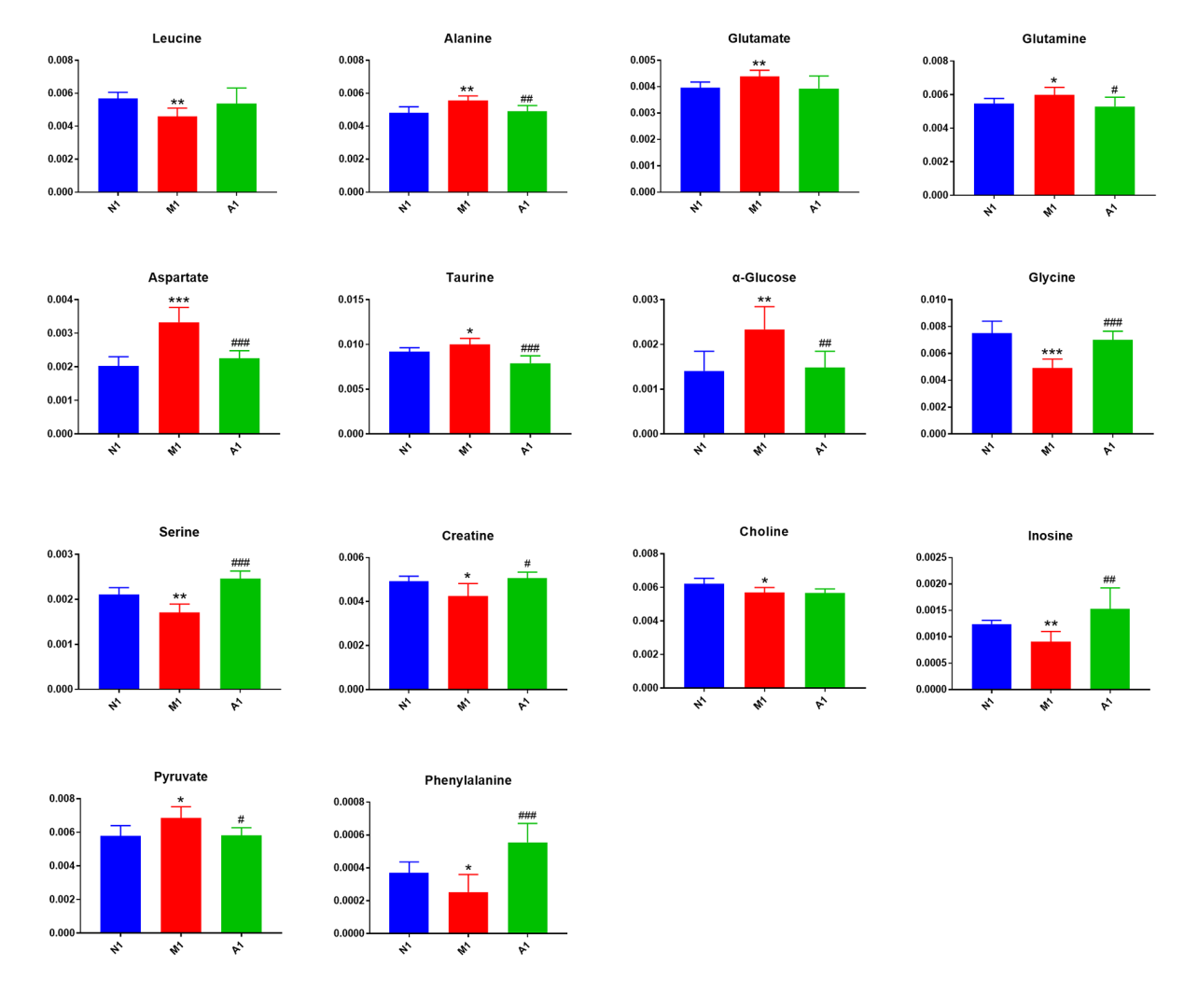


**Fig. S2** Relative abundance (mean ± SD) of characteristic metabolites from medulla tissues of rats in the T1 subgroup of Control (blue), GML (red) and EA (green) group. ^***^*P*<0.001, ^**^*P*<0.01, ^*^*P*<0.05 compared with the control group; ^###^*P*<0.001, ^##^*P*<0.01, ^#^*P*<0.05 compared with the GML group.


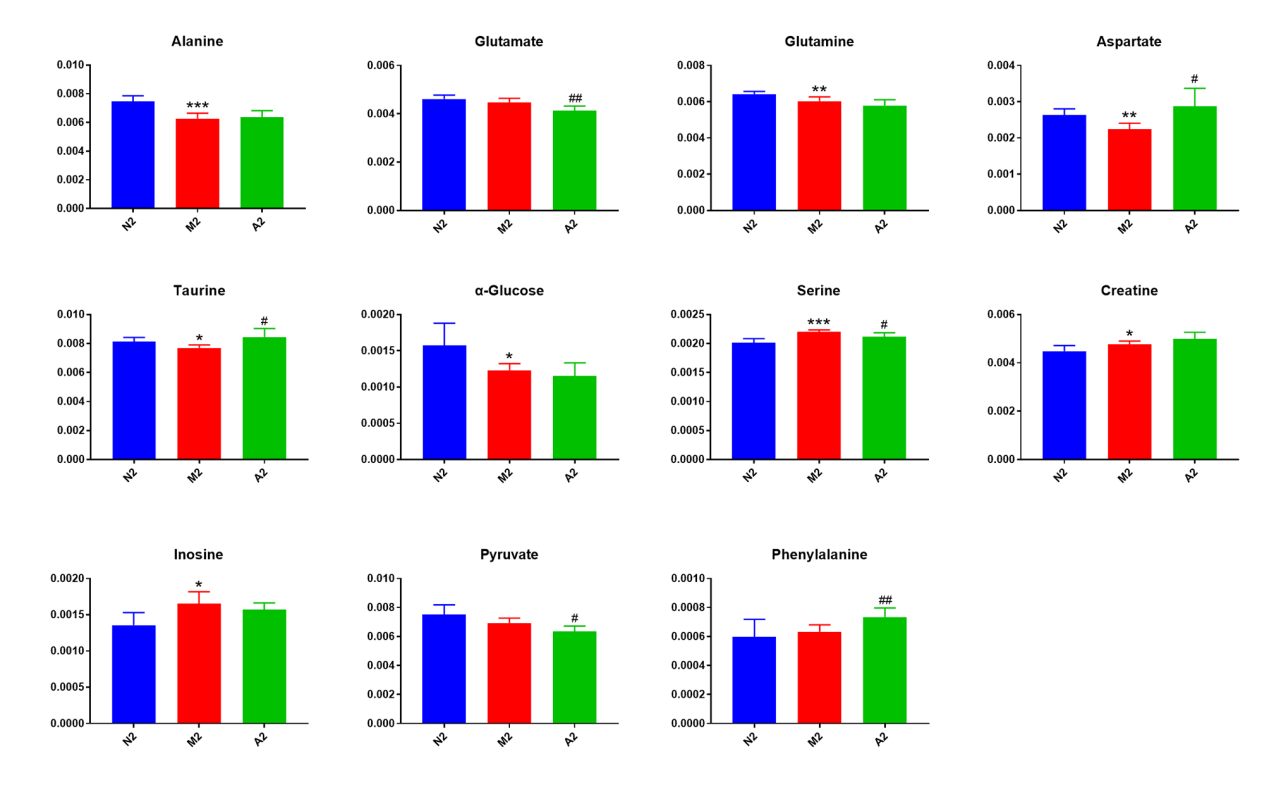


**Fig. S3** Relative abundance (mean ± SD) of characteristic metabolites from medulla tissues of rats in the T4 subgroup of Control (blue), GML (red) and EA (green) group. ^***^*P*<0.001, ^**^*P*<0.01, ^*^*P*<0.05 compared with the control group; ^###^*P*<0.001, ^##^*P*<0.01, ^#^*P*<0.05 compared with the GML group.


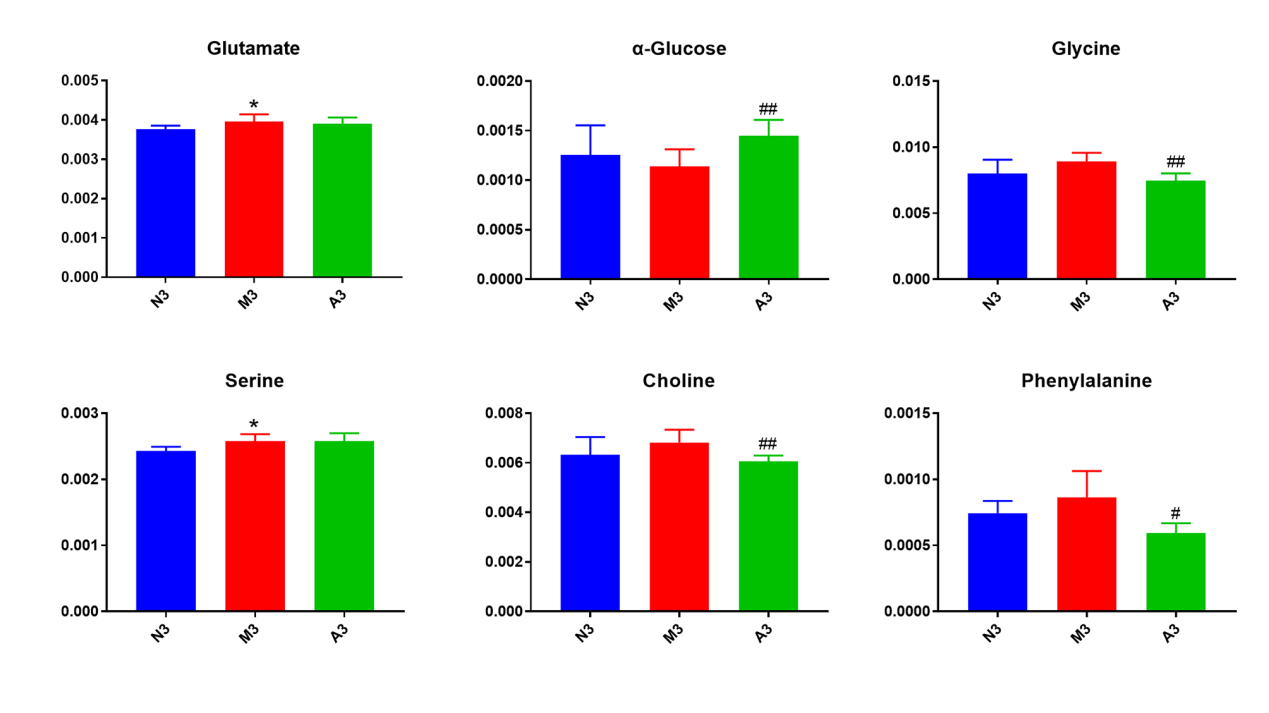


**Fig. S4** Relative abundance (mean ± SD) of characteristic metabolites from medulla tissues of rats in the T7 subgroup of Control (blue), GML (red) and EA (green) group. ^***^*P*<0.001, ^**^*P*<0.01, ^*^*P*<0.05 compared with the control group; ^###^*P*<0.001, ^##^*P*<0.01, ^#^*P*<0.05 compared with the GML group.


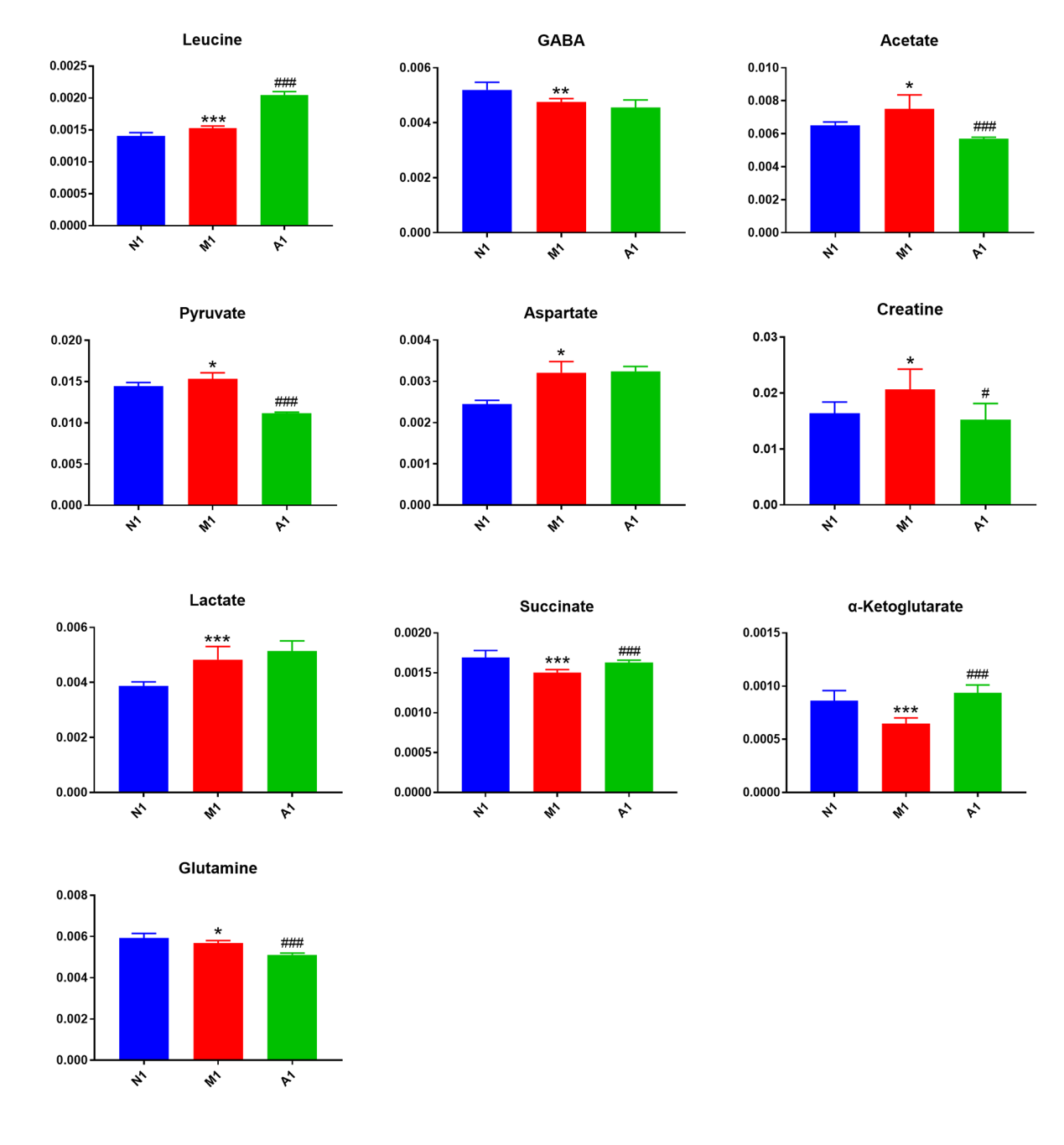


**Fig. S5** Relative abundance (mean ± SD) of characteristic metabolites from cerebral cortex tissues of rats in the T1 subgroup of Control (blue), GML (red) and EA (green) group. ^***^*P*<0.001, ^**^*P*<0.01, ^*^*P*<0.05 compared with the control group; ^###^*P*<0.001, ^##^*P*<0.01, ^#^*P*<0.05 compared with the GML group.


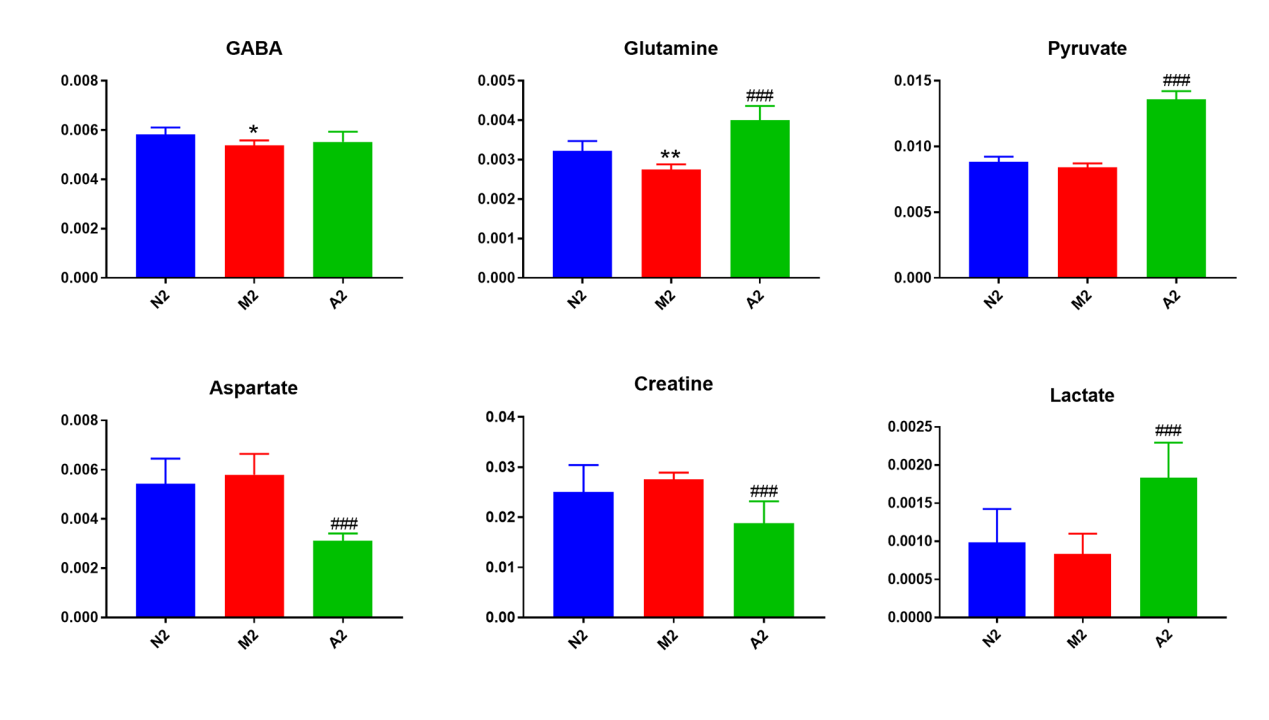


**Fig. S6** Relative abundance (mean ± SD) of characteristic metabolites from cerebral cortex tissues of rats in the T4 subgroup of Control (blue), GML (red) and EA (green) group. ^***^*P*<0.001, ^**^*P*<0.01, ^*^*P*<0.05 compared with the control group; ^###^*P*<0.001, ^##^*P*<0.01, ^#^*P*<0.05 compared with the GML group.


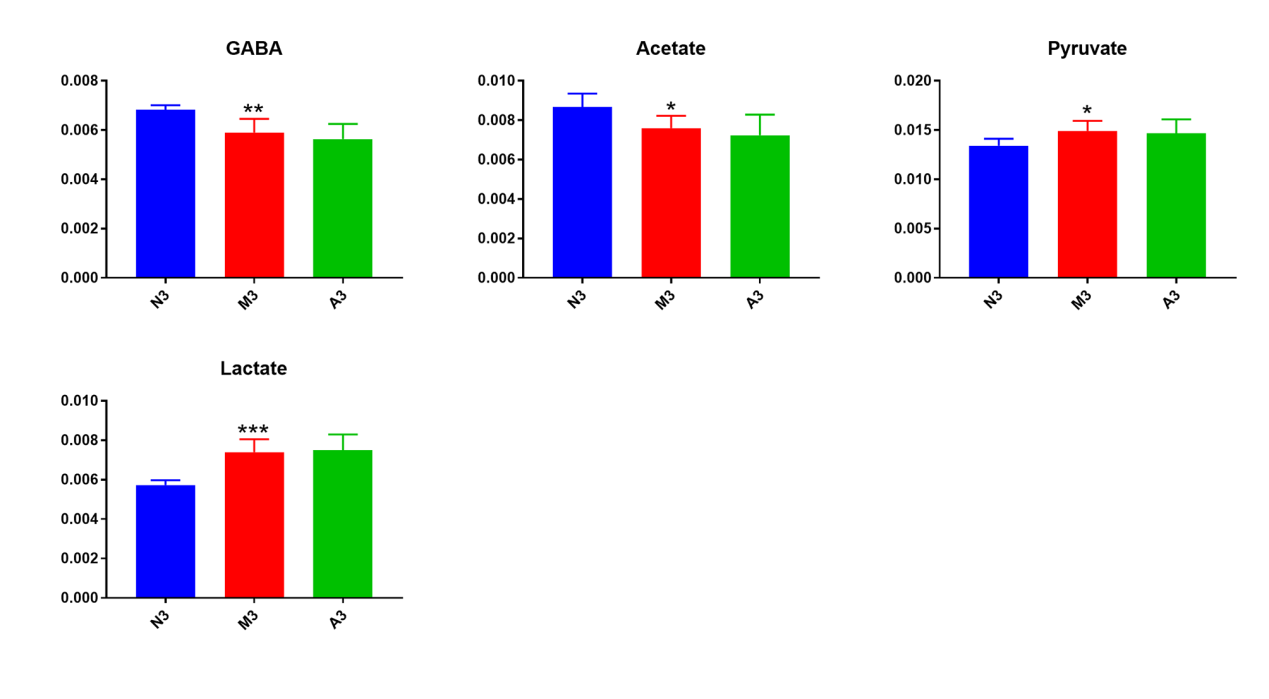


**Fig. S7** Relative abundance (mean ± SD) of characteristic metabolites from cerebral cortex tissues of rats in the T7 subgroup of Control (blue), GML (red) and EA (green) group. ^***^*P*<0.001, ^**^*P*<0.01, ^*^*P*<0.05 compared with the control group; ^###^*P*<0.001, ^##^*P*<0.01, ^#^*P*<0.05 compared with the GML group.

**Table S1** Peak attribution of the main marked metabolites in ^1^H-NMR spectra of stomach sample.

| **NO.** | **Metabolites** | **δ1H/ppm** | **Moieties** | **Sample** |
| --- | --- | --- | --- | --- |
| 1 | LDL | 0.86(br) | CH3-(CH2)n- | St |
| 2 | VLDL | 0.89(br) | CH3-(CH2)n- | St |
| 3 | Isoleucine | 0.94(t); 1.01(d) | δ-CH3; β-CH3 | St |
| 4 | Leucine | 0.96(t); 1.70(m) | CH3; CH2&γ-CH | St |
| 5 | Valine | 0.99(d); 1.04(d) | γ-CH3; γ-CH´3 | St |
| 6 | OIV | 1.12(d) | CH3 | St |
| 7 | Ethanol | 1.19(t);3.66(q) | CH3; CH2 | St |
| 8 | Methylmalonate | 1.23(d) | CH3 | St |
| 9 | Lactate | 1.33(d); 4.11(q) | CH3; CH | St |
| 10 | Alanine | 1.48(d); 3.78(q) | CH3; CH | St |
| 11 | Lysine | 1.73(m);1.88(m);3.03(t) | γ-CH2; δ-CH2;β-CH2; ε-CH2; α-CH | St |
| 12 | GABA | 1.90(m);2.30(t);3.02(t) | α-CH2; β-CH2 | St |
| 13 | Acetate | 1.92(s) | CH3 | St |
| 14 | NAA | 2.02(s);2.50(m) | CH3 | St |
| 15 | Glutamate | 2.05(m);2.13(m);2.35(m) | β-CH | St |
| 16 | Glutamine | 2.14(m);2.46(m) | β-CH2 | St |
| 17 | Glutathione | 2.17(m); 2.55(m) | β-CH2; γ-CH2 | St |
| 18 | Pyruvate | 2.37(s) | CH3 | St |
| 19 | Oxaloacetate | 2.38(s) | CH2 | St |
| 20 | Succinate | 2.41(s) | CH | St |
| 21 | α-Ketoglutarate | 2.45(t); 3.01(t) | γ-CH2; β-CH2 | St |
| 22 | Citrate | 2.54(d); 2.69(d) | CH2 | St |
| 23 | Sarcosine | 2.74(s);3.60(s) | CH3; CH2 | St |
| 24 | Aspartate | 2.67(dd);2.82(dd);3.90(dd) | β-CH; β-CH' | St |
| 25 | DMG | 2.93(s);3.73(s) | CH3; CH2 | St |
| 26 | Creatine | 3.04(s); 3.93(s) | CH3; CH2 | St |
| 27 | Creatinine | 3.05(s); 4.06(s) | CH3; CH2 | St |
| 28 | Phenylalanine | 3.13(m);3.28(m);7.33(d);7.38(t) | β-CH´; o-CH; p-CH | St |
| 29 | Ethanolamine | 3.14(t); 3.84(t) | CH2NH2; CH2OH | St |
| 30 | Choline | 3.20(s);3.52(m);4.07(m) | CH3;N-CH2;O-CH2 | St |
| 31 | Phosphocholine | 3.22(s);3.59(m);4.17(m) | CH3;N-CH2;O-CH2 | St |
| 32 | GPC | 3.23(s); 3.96(m) | CH3; CH&O-CH2 | St |
| 33 | β-Glucose | 3.25(dd);3.51(t);3.46(m); 3.49(t);3.72(dd);3.90(dd); 4.65(d) | CH(2);CH(4);CH(5); CH(3);CH(6);CH(6’); CH(1) | St |
| 34 | Taurine | 3.27(t);3.43(t) | CH3; α-CH; β-CH | St |
| 35 | Myo-inositol | 3.28(t);3.54(dd);3.63(t); 4.07(t) | CH(2);CH(4,6);CH(1,3); CH(5) | St |
| 36 | Methanol | 3.37(s) | CH3 | St |
| 37 | α-Glucose | 3.42(t);3.54(dd);3.71(t);3.74(m);3.84(m);5.24(d) | CH(4);CH(2);CH(3); CH(6);CH(5&6); CH(1); | St |
| 38 | Glycine | 3.56(s) | CH2 | St |
| 39 | Glycerol | 3.57(m);3.62(m); 3.79(m) | CH2; CH'2; CH | St |
| 40 | Glycogen | 3.63(m);3.83(m);3.96(m) | CH(3,5,29);CH(6,23,41); CH(4,9); CH(2,17,25) | St |
| 41 | Guanidoacetate | 3.80(s) | CH2 | St |
| 42 | Serine | 3.83(dd); 3.96(m) | CH; CH2 | St |
| 43 | Hippurate | 3.97(d);7.84(d) | CH2;CH(3,5);CH(4);CH(2,6) | St |
| 45 | Inosine | 4.28(dd);4.44(t);6.09(d);8.22(s); 8.34(s) | CH(5); N-CH=N | St |
| 46 | Adenosine | 4.30(dd); 8.26(s); 8.35(s) | CH(5); N-CH=N | St |
| 47 | Allantoin | 5.39(s) | CH | St |
| 48 | U-Glc | 5.61(dd);5.98(m);7.96(d) | CH(2);CH(21,31);CH(32) | St |
| 49 | Uracil | 5.80(d); 7.53(d) | CH(5); CH(6) | St |
| 50 | Cytidine | 5.89(d);6.06(d);7.83(d) | CH(10); CH(2); CH(11) | St |
| 51 | Uridine | 5.90(d); 7.87(d) | CH(10); CH(11) | St |
| 52 | Fumarate | 6.52(s) | CH | St |
| 53 | Tyrosine | 6.89(d); 7.19(d) | m-CH; o-CH | St |
| 54 | 3-Methylhistidine | 7.04(s); 7.68(s) | CH(2); CH(4) | St |
| 55 | Histidine | 7.08(s);7.83(s) | β-CH;CH(5);CH(2); | St |
| 56 | Xanthine | 7.93(s) | CH | St |
| 57 | Nicotinamide | 8.24(dd);8.72(dd); 8.94(s) | CH(4);CH(6);CH(2) | St |

s: singlet, d: doublet, t: triplet, q: quartet, m: multiplet, dd: doublet of doublet, St: stomach.

**Table S2** Peak attribution of the main marked metabolites in ^1^H-NMR spectra of medulla sample.

| **NO.** | **Metabolites** | **δ1H/ppm** | **Moieties** | **Sample** |
| --- | --- | --- | --- | --- |
| 1 | LDL | 0.86(br) | CH3-(CH2)n- | M |
| 2 | VLDL | 0.89(br) | CH3-(CH2)n- | M |
| 3 | Isoleucine | 0.94(t); 1.01(d) | δ-CH3; β-CH3 | M |
| 4 | Leucine | 0.96(t); 1.70(m) | CH3; CH2&γ-CH | M |
| 5 | Valine | 0.99(d); 1.04(d) | γ-CH3; γ-CH´3 | M |
| 6 | OIV | 1.12(d) | CH3 | M |
| 7 | Ethanol | 1.19(t);3.66(q) | CH3; CH2 | M |
| 8 | Methylmalonate | 1.23(d) | CH3 | M |
| 9 | Lactate | 1.33(d); 4.11(q) | CH3; CH | M |
| 10 | Alanine | 1.48(d); 3.78(q) | CH3; CH | M |
| 11 | Lysine | 1.73(m);1.88(m);3.03(t) | γ-CH2; δ-CH2;β-CH2; ε-CH2; α-CH | M |
| 12 | GABA | 1.90(m);2.30(t);3.02(t) | α-CH2; β-CH2 | M |
| 13 | Acetate | 1.92(s) | CH3 | M |
| 14 | NAA | 2.02(s);2.50(m) | CH3 | M |
| 15 | Glutamate | 2.05(m);2.13(m);2.35(m) | β-CH | M |
| 16 | Glutamine | 2.14(m);2.46(m) | β-CH2 | M |
| 17 | Glutathione | 2.17(m); 2.55(m) | β-CH2; γ-CH2 | M |
| 18 | Pyruvate | 2.37(s) | CH3 | M |
| 19 | Oxaloacetate | 2.38(s) | CH2 | M |
| 20 | Succinate | 2.41(s) | CH | M |
| 21 | α-Ketoglutarate | 2.45(t); 3.01(t) | γ-CH2; β-CH2 | M |
| 22 | Citrate | 2.54(d); 2.69(d) | CH2 | M |
| 24 | Aspartate | 2.67(dd);2.82(dd);3.90(dd) | β-CH; β-CH' | M |
| 25 | DMG | 2.93(s);3.73(s) | CH3; CH2 | M |
| 26 | Creatine | 3.04(s); 3.93(s) | CH3; CH2 | M |
| 30 | Choline | 3.20(s);3.52(m);4.07(m) | CH3;N-CH2;O-CH2 | M |
| 31 | Phosphocholine | 3.22(s);3.59(m);4.17(m) | CH3;N-CH2;O-CH2 | M |
| 34 | Taurine | 3.27(t);3.43(t) | CH3; α-CH; β-CH | M |
| 35 | Myo-inositol | 3.28(t);3.54(dd);3.63(t); 4.07(t) | CH(2);CH(4,6);CH(1,3); CH(5) | M |
| 36 | Methanol | 3.37(s) | CH3 | M |
| 37 | α-Glucose | 3.42(t);3.54(dd);3.71(t);3.74(m);3.84(m);5.24(d) | CH(4);CH(2);CH(3); CH(6);CH(5&6); CH(1); | M |
| 38 | Glycine | 3.56(s) | CH2 | M |
| 39 | Glycerol | 3.57(m);3.62(m); 3.79(m) | CH2; CH'2; CH | M |
| 42 | Serine | 3.83(dd); 3.96(m) | CH; CH2 | M |
| 44 | Phosphocreatine | 3.93(s) | CH2 | M |
| 45 | Inosine | 4.28(dd);4.44(t);6.09(d);8.22(s); 8.34(s) | CH(5); N-CH=N | M |
| 46 | Adenosine | 4.30(dd); 8.26(s); 8.35(s) | CH(5); N-CH=N | M |
| 47 | Allantoin | 5.39(s) | CH | M |
| 49 | Uracil | 5.80(d); 7.53(d) | CH(5); CH(6) | M |
| 51 | Uridine | 5.90(d); 7.87(d) | CH(10); CH(11) | M |
| 52 | Fumarate | 6.52(s) | CH | M |
| 53 | Tyrosine | 6.89(d); 7.19(d) | m-CH; o-CH | M |
| 54 | 3-Methylhistidine | 7.04(s); 7.68(s) | CH(2); CH(4) | M |
| 55 | Histidine | 7.08(s);7.83(s) | β-CH;CH(5);CH(2); | M |
| 56 | Xanthine | 7.93(s) | CH | M |
| 57 | Nicotinamide | 8.24(dd);8.72(dd); 8.94(s) | CH(4);CH(6);CH(2) | M |
| 58 | Formate | 8.46(s) | CH | M |

s: singlet, d: doublet, t: triplet, q: quartet, m: multiplet, dd: doublet of doublet, M: medulla.

**Table S3** Peak attribution of the main marked metabolites in ^1^H-NMR spectra of cerebral cortex sample.

| **NO.** | **Metabolites** | **δ1H/ppm** | **Moieties** | **Sample** |
| --- | --- | --- | --- | --- |
| 3 | Isoleucine | 0.94(t); 1.01(d) | δ-CH3; β-CH3 | CC |
| 4 | Leucine | 0.96(t); 1.70(m) | CH3; CH2&γ-CH | CC |
| 5 | Valine | 0.99(d); 1.04(d) | γ-CH3; γ-CH´3 | CC |
| 7 | Ethanol | 1.19(t);3.66(q) | CH3; CH2 | CC |
| 8 | Methylmalonate | 1.23(d) | CH3 | CC |
| 9 | Lactate | 1.33(d); 4.11(q) | CH3; CH | CC |
| 10 | Alanine | 1.48(d); 3.78(q) | CH3; CH | CC |
| 11 | Lysine | 1.73(m);1.88(m);3.03(t) | γ-CH2; δ-CH2;β-CH2; ε-CH2; α-CH | CC |
| 12 | GABA | 1.90(m);2.30(t);3.02(t) | α-CH2; β-CH2 | CC |
| 13 | Acetate | 1.92(s) | CH3 | CC |
| 14 | NAA | 2.02(s);2.50(m) | CH3 | CC |
| 15 | Glutamate | 2.05(m);2.13(m);2.35(m) | β-CH | CC |
| 16 | Glutamine | 2.14(m);2.46(m) | β-CH2 | CC |
| 18 | Pyruvate | 2.37(s) | CH3 | CC |
| 19 | Oxaloacetate | 2.38(s) | CH2 | CC |
| 20 | Succinate | 2.41(s) | CH | CC |
| 21 | α-Ketoglutarate | 2.45(t); 3.01(t); | γ-CH2; β-CH2 | CC |
| 22 | Citrate | 2.54(d); 2.69(d) | CH2 | CC |
| 23 | Sarcosine | 2.74(s);3.60(s) | CH3; CH2 | CC |
| 24 | Aspartate | 2.67(dd);2.82(dd);3.90(dd) | β-CH; β-CH' | CC |
| 25 | DMG | 2.93(s);3.73(s) | CH3; CH2 | CC |
| 26 | Creatine | 3.04(s); 3.93(s) | CH3; CH2 | CC |
| 27 | Creatinine | 3.05(s); 4.06(s) | CH3; CH2 | CC |
| 28 | Phenylalanine | 3.13(m);3.28(m);7.33(d);7.38(t) | β-CH´; o-CH; p-CH | CC |
| 30 | Choline | 3.20(s);3.52(m);4.07(m) | CH3;N-CH2;O-CH2 | CC |
| 31 | Phosphocholine | 3.22(s);3.59(m);4.17(m) | CH3;N-CH2;O-CH2 | CC |
| 32 | GPC | 3.23(s); 3.96(m) | CH3; CH&O-CH2 | CC |
| 33 | β-Glucose | 3.25(dd);3.51(t);3.46(m); 3.49(t);3.72(dd);3.90(dd); 4.65(d) | CH(2);CH(4);CH(5); CH(3);CH(6);CH(6’); CH(1) | CC |
| 34 | Taurine | 3.27(t);3.43(t) | CH3; α-CH; β-CH | CC |
| 35 | Myo-inositol | 3.28(t);3.54(dd);3.63(t); 4.07(t) | CH(2);CH(4,6);CH(1,3); CH(5) | CC |
| 36 | Methanol | 3.37(s) | CH3 | CC |
| 37 | α-Glucose | 3.42(t);3.54(dd);3.71(t);3.74(m);3.84(m);5.24(d) | CH(4);CH(2);CH(3); CH(6);CH(5&6); CH(1) | CC |
| 38 | Glycine | 3.56(s) | CH2 | CC |
| 39 | Glycerol | 3.57(m);3.62(m);3.79(m) | CH2; CH'2; CH | CC |
| 40 | Glycogen | 3.63(m);3.83(m);3.96(m) | CH(3,5,29);CH(6,23,41); CH(4,9); CH(2,17,25) | CC |
| 41 | Guanidoacetate | 3.80(s) | CH2 | CC |
| 42 | Serine | 3.83(dd); 3.96(m) | CH; CH2 | CC |
| 44 | Phosphocreatine | 3.93(s) | CH2 | CC |
| 45 | Inosine | 4.28(dd);4.44(t);6.09(d);8.22(s); 8.34(s) | CH(5); N-CH=N | CC |
| 46 | Adenosine | 4.30(dd); 8.26(s); 8.35(s) | CH(5); N-CH=N | CC |
| 47 | Allantoin | 5.39(s) | CH | CC |
| 48 | U-Glc | 5.61(dd);5.98(m);7.96(d) | CH(2);CH(21,31);CH(32) | CC |
| 49 | Uracil | 5.80(d); 7.53(d) | CH(5); CH(6) | CC |
| 50 | Cytidine | 5.89(d);6.06(d);7.83(d) | CH(10); CH(2); CH(11) | CC |
| 52 | Fumarate | 6.52(s) | CH | CC |
| 53 | Tyrosine | 6.89(d); 7.19(d) | m-CH; o-CH | CC |
| 54 | 3-Methylhistidine | 7.04(s); 7.68(s) | CH(2); CH(4) | CC |
| 55 | Histidine | 7.08(s);7.83(s) | β-CH;CH(5);CH(2) | CC |
| 56 | Xanthine | 7.93(s) | CH | CC |
| 57 | Nicotinamide | 8.24(dd);8.72(dd); 8.94(s) | CH(4);CH(6);CH(2) | CC |
| 58 | Formate | 8.46(s) | CH | CC |

s: singlet, d: doublet, t: triplet, q: quartet, m: multiplet, dd: doublet of doublet, CC: cerebral cortex
